# Prediction model for dengue fever based on interactive effects between multiple meteorological factors in Guangdong, China (2008–2016)

**DOI:** 10.1371/journal.pone.0225811

**Published:** 2019-12-09

**Authors:** Binghua Zhu, Ligui Wang, Haiying Wang, Zhidong Cao, Lei Zha, Ze Li, Zhongyang Ye, Jinping Zhang, Hongbin Song, Yansong Sun

**Affiliations:** 1 Chinese PLA Center for Disease Control and Prevention, Beijing, China; 2 305 Hospital of PLA, Beijing, China; 3 Joint Service Institute, National Defense University of PLA, Beijing, China; 4 The State Key Laboratory of Management and Control for Complex Systems, Institute of Automation, Chinese Academy of Sciences, Beijing, China; 5 College of Military Medicine, Academy of Military Sciences, Beijing, China; The University of Hong Kong, CHINA

## Abstract

**Introduction:**

In order to improve the prediction accuracy of dengue fever incidence, we constructed a prediction model with interactive effects between meteorological factors, based on weekly dengue fever cases in Guangdong, China from 2008 to 2016.

**Methods:**

Dengue fever data were derived from statistical data from the China National Notifiable Infectious Disease Reporting Information System. Daily meteorological data were obtained from the China Integrated Meteorological Information Sharing System. The minimum temperature for transmission was identified using data fitting and the Ross-Macdonald model. Correlations and interactive effects were examined using Spearman’s rank correlation and multivariate analysis of variance. A probit regression model to describe the incidence of dengue fever from 2008 to 2016 and forecast the 2017 incidence was constructed, based on key meteorological factors, interactive effects, mosquito-vector factors, and other important factors.

**Results:**

We found the minimum temperature suitable for dengue transmission was ≥18°C, and as 97.91% of cases occurred when the minimum temperature was above 18 °C, the data were used for model training and construction. Epidemics of dengue are related to mean temperature, maximum/minimum and mean atmospheric pressure, and mean relative humidity. Moreover, interactions occur between mean temperature, minimum atmospheric pressure, and mean relative humidity. Our weekly probit regression prediction model is 0.72. Prediction of dengue cases for the first 41 weeks of 2017 exhibited goodness of fit of 0.60.

**Conclusion:**

Our model was accurate and timely, with consideration of interactive effects between meteorological factors.

## Introduction

Dengue fever is an acute infectious disease caused by the dengue virus and transmitted by the *Aedes* mosquito. It is a serious public health issue in tropical and subtropical zones. According to the World Health Organisation, the incidence of dengue fever has risen sharply over the past few decades. More than 40% of the global population is at risk of dengue fever, with 50–100 million cases of dengue infections each year[1]. Guangdong is a coastal province on the southern tip of China, experiences frequent population movement with Southeast Asian countries, and possesses a subtropical monsoon climate. These conditions are ideal for the breeding of the dengue vector (*Aedes* mosquito) and the importation of foreign dengue cases[2]. The first reported outbreak of dengue fever in China occurred in May 1978 in Foshan, Guangdong[3]. Since then, Guangdong has become the main dengue epidemic area in China. From 2008 to 2014, the percentage of the total dengue fever cases in China that occurred in Guangdong increased from 34.25% to 96.12%[4]. Dengue fever is a serious threat to people's health. Therefore, exploring factors influencing the dengue outbreaks in Guangdong and predicting future epidemiological trends are of great significance to the prevention and control of dengue fever.

Previous studies regarding the epidemiological patterns of dengue fever have focused on the impact of meteorological factors, imported cases, and control measures. However, most studies used monthly or yearly data, and did not consider the interactive effects among meteorological factors, leading to unreliable or even contradictory results. Li et al analysed the epidemiological distribution of dengue fever and the relationship with meteorological factors based on monthly data from Guangzhou in 2007 to 2012 and reported that relative humidity was positively correlated with dengue epidemics[5]. In contrast, when Minh et al analysed the monthly data of dengue epidemics in Hanoi from 2002 to 2010, they found that relative humidity was negatively correlated with dengue epidemics[6].Moreover, due to a large time granularity, this led to a low goodness of fit. For example, the goodness of fit of the binomial regression model by Adde et al was 0.88[7], while the SARIMA model constructed by Gharbiet et al was 0.72[8]. These contradictory resultscan also be observed in studies from Fan et al[9], Tang et al[10], and Xiao et al[11].

Therefore, it is especially important to perform correlation analyses between dengue fever and meteorological factors based on a small time granularity and to clarify which meteorological factors play a decisive role in dengue epidemics. It is also important to examine the interactive effects of different meteorological factors on dengue epidemics. Based on the above multiple variables, such as key meteorological factors, interactive meteorological factors, mosquito-vector factors, and importation factors, we aimed to establish an accurate prediction model and to provide support for dengue prediction and warnings.

## Methods

### Ethics statement

This experimental protocol was approved and authorised by the Academy of Military Medical Sciences Review Board. Dengue data comes from statistical data from the China National Notifiable Infectious Disease Reporting Information System and did not involve identifiable data. There was no request for ethical permission, according to Chinese law. Methods were carried out in accordance with the relevant institutional guidelines.

### Source of data

Daily dengue fever cases and patient information from 2008 to 2016 in Guangdong Province were obtained from the China National Notifiable Infectious Disease Reporting Information System (NNDS) (http://www.chinacdc.cn/). General physicians are required to diagnose the disease according to the National Diagnostic Criteria for Dengue Fever (WS216-2008) and report new cases to the web-based NNDS within 24 hours. Using annual calendar weeks as the unit, the sum of the daily cases in a week was used to determine the number of weekly cases.

Daily meteorological data were obtained from the China Integrated Meteorological Information Sharing System (http://data.cma.cn/). Nine meteorological factors were collected, including mean temperature, maximum temperature, minimum temperature, mean atmospheric pressure, maximum atmospheric pressure, minimum atmospheric pressure, mean relative humidity, maximum wind speed, and extreme wind speed. Using annual calendar weeks as the unit, daily meteorological data were summed, and weekly averages were calculated. Population data were obtained from the Statistics Bureau of Guangdong Province (http://www.gdstats.gov.cn/).

### Optimal minimum temperature selection

The minimum temperature has an important influence on the survival of mosquitoes and the epidemic of dengue[11–13]. Therefore, using the data above minimum temperature suitable for dengue fever reduces the data noise and improves the data quality of the model. Based on the Ross-Macdonald[14]and Watts[15]models, we searched for the optimal minimum temperature suitable for dengue transmission. In this model, infectious life P^n^/(−1nP)≥1day can be used as the minimum temperature suitable for dengue transmission, where P is the daily survival probability. 1/−1nP is the expected life, and P^n^ is the probability of survival of infected mosquitoes after n days; n is mainly determined by the temperature, with a relationship of n = K/(T−C) [15], where K is the effective accumulated temperature(165.2 °C) needed for the incubation of dengue virus in mosquitoes and C is the minimum temperature (11.8°C)needed for the incubation of dengue viruses in mosquitoes and T is the minimum temperature required for a mosquito to survive for at least one day. Therefore, n = 165.2/(T−11.8). The daily survival probability, such as P = 0.85[16]or 0.89[17], was obtained under laboratory conditions with constant temperature, humidity, and light, whereas daily survival probability obtained under natural environments were 0.913 in French Guiana[18] and 0.918in Macao[19]. To calculate the minimum temperature suitable for the transmission of dengue fever, we selected the daily survival probability for Macao of P = 0.918, as it is adjacent to Guangdong province.

In order to explore the relationship between dengue fever and temperature, we divided the average weekly minimum temperature into 2 °C intervals of 8–10 °C, 10–12 °C, 12–14 °C, 14–16 °C, 16–18 °C, 18–20 °C, 20–22 °C, 22–24 °C, and 24–26 °C. The cumulative numbers of cases corresponding to these intervals were then calculated, and the moving smooth curve was used to determine the optimal temperature of dengue fever epidemics. For the different temperature intervals, the corresponding number of cases were 13, 54, 95, 156, 709, 955, 17,956, 17,894, and 13,298, respectively.

### Correlation and interactive effects analysis of meteorological factors

Among the 282 weeks of data for 2008–2016, Spearman rank correlation was used to analyse the correlation of the meteorological factors with dengue fever epidemics occurring above the suitable temperature. Multivariate analysis of variance was used to determine interactive effects among meteorological factors. Spearman rank correlation and multivariate analysis of variance were carried out using SPSS 19.0 statistical package. P < 0.05 was considered statistically significant.

Further analysis was performed to explore if there were interactive effects among the various meteorological factors related with dengue fever epidemics, when the minimum temperature was ≥18°C. Continuous meteorological factors were converted into categorical meteorological factors according to quartiles. Multivariate analysis of variance was performed to comprehensively consider the differences in the number of cases under multiple meteorological conditions.

### Prediction model based on multiple factors

The inverse cumulative normal distribution model based on meteorological factors, mosquito-vector factors, and importation factors is given as follows:

$$Norminv\left(\frac{c}{N}\right)=\beta_{0}+\sum_{\text{k}=1}^{\text{n}+2}\beta_{k}x_{k}$$ (1)

where c is the number of cases, N is the population size, n is the number of key meteorological factors, β_0_ is the model construction constant, and β_k_ and X_k_ are the variable and variable coefficients.

The maximum likelihood estimation (MLE)was used for parameter estimation in the inverse cumulative normal distribution model:

The key meteorological factors correlated to dengue epidemics and the interactive meteorological factors were labelled as *x*_*1*_, *x*_*2*_, *x*_*3*_, ……*x*_*n*_, respectively. The Breteau Index is an index for evaluating the density of *Aedes* mosquitoes in a region, that is, the average number of containers for the breeding of *Aedes* larvae per 100 households. If the Breteau Index is below 5, it is safe; if it is above 20, it means that once external cases are imported, an epidemic of mosquito-borne infectious diseases may occur in this area. Dengue epidemic risk is related to the Breteau Index, which is closely related to meteorological factors. In this study, due to the lack of Breteau Index, a model of mosquito vectors [20–22]was constructed based on temperature, which waslabelled as *x*_*n+1*_. This assumes that there is an optimum temperature, α2, that gives the maximum probability of vector breeding and reproduction, and hence has the maximum density. Each time the temperature deviates from α_3_, the vector density is reduced by 1/α_1_. Hence, for a given mean temperature, *x*_*1*_, the corresponding mosquito-vector factor is as follows:

$$x_{n+1}=\frac{1}{a_{1}\left(|x_{1}-a_{2}|/a_{3}\right)}$$ (2)

where α_1_, α_2_, and α_3_ refer to the rate of vector density decay, the rational temperature value for vector breeding, and the step size of vector density decay, respectively. However, since the independent mosquito-vector factor, *x*_*n+1*_, contained three unknown parameters, the simulated annealing algorithm was applied to obtain the optimal estimate of the mosquito-vector parameter set (α_1_, α_2_, α_3_).

The observed number of new dengue fever cases per week was *obs*_*i*_(*i* = 1,2,3,…,N), and the estimation obtained from the logit model was *est*_*i*_(*i* = 1,2,3,…,N). Hence, the objective function of MLE, *loss*, which is also the R^2^ (goodness of fit), was calculated as follows:

$$loss=R^{2}=1-\frac{\sum_{i=1}^{N}{\left(obs_{i}-est_{i}\right)}^{2}}{\sum_{i=1}^{N}{\left(obs_{i}-\sum_{i=1}^{N}\frac{obs_{i}}{N}\right)}^{2}}$$ (3)

By comparing the optimal solution with the actual observation value, the loss value corresponding to (α_1_, α_2_, α_3_) can be obtained by using formula (3). If the loss value meets the criteria (this loss value is better than the previous loss value, or no better loss can be found after 300 repetitions), then this (α_1_, α_2_, α_3_) is the approximate optimal solution. If it does not meet the requirements, a new (α_1_, α_2_, α_3_) is generated by random perturbation (α_1_, α_2_, α_3_). The above process is repeated to obtain a new loss value and to check whether it meets the requirements.

The steps for estimating the model parameters β_k_ were as follows:

Step 1: Arbitrarily specify the control parameter set P = {α_1_, α_2_, α_3_}, then use MLE to obtain the optimal estimate of the logit model and generate the predicted value of new dengue fever cases per week that corresponds to the parameter P.
Step 2: Use the formula for calculating *loss* above to obtain the objective function value *loss* in the forecasted data and observed data of new dengue fever cases per week.
Step 3: Apply random perturbation to the control parameter set P (select 1 of the parameters randomly and add to it a random perturbation value that follows the normal distribution, keeping other parameter values unchanged) and obtain a new control parameter set P′. Calculate the value of the objective function *loss*′ corresponding to P′.
Step 4: Replace P with P′ according to the Metropolis rule:
  $$\left\{ \begin{array}{ll} p\left(p\to p^{\prime }\right)=1 & loss\prime \leq loss\\ p\left(p\to p^{\prime }\right)=exp\left(\frac{loss-loss^{\prime }}{c}\right) & loss\prime >loss \end{array}\right.$$ (4)
  where *p*(P→P′) indicates the probability of P′ replacing P. The temperature parameter *c* is a variable; during loop iteration, each time the probability selection of *loss*′>*loss* is performed, *c* decreases by a certain proportion (i.e., *c* = *k × c*, where *k* is a constant temperature drop parameter less than 1).
Step 5: When the number of invalid continuous perturbations is greater than the set threshold, the iteration ends. The control parameter set P prior to the last invalid perturbance becomes the estimation result for the final approximate optimal control parameter.

Due to the large number of imported cases from the Southeast Asia dengue epidemic, there was a major dengue outbreak in Guangdong in 2014. Therefore, the importation factor was added to the model, which was labelled as *x*_*n+2*_ and was a binary variable, where 2014 was assigned a value of 1 and all other years were assigned a value of 0.

$$x_{n+2}=\{\begin{array}{cc} 1 & year=2014\\ 0 & otherwise \end{array}$$ (5)

The goodness of fit index, R^2^, can be used to measure the overall effect of the model's prediction. The value range is (−∞, 1) and the closer the value is to 1, the better the predictive effect of the model, and vice versa. Goodness of fit indicates a good fit between the predicted and actual values, whereas the correlation coefficient indicates a consistent trend between the predicted and actual value.

## Results

### Optimal minimum temperature

The minimum temperature was determined when given the infectious life of infected mosquitoes and daily survival probability, P, of *Aedes* mosquitoes (Table 1). When the daily survival probability of *Aedes* mosquitoes was 0.850, 0.890, 0.913, and 0.918, given that the infectious life of infected mosquitoes was ≥1 day, the minimum temperatures were 27°C, 21°C, 19°C, and 18°C, respectively. As Guangdong is geographically close to Macao, these locations share similar meteorological factors and mosquito habits. Therefore, we selected 0.918 for the *Aedes* mosquitoes in Macaoas as the daily survival probability of Guangdong. When the infectious life of a mosquito is at least one day (i.e. 1.196), the corresponding minimum temperature is 18°C, as shown in Table 1.

**Table 1:** Mean duration of the infectious period (days) at different minimum temperature and daily survival probability.

Daily survival probability		Mean duration of the infectious period (days)										
	T (°C):	17	18	19	20	21	22	23	24	25	26	27
0.850		0.035	0.081	0.148	0.233	0.332	0.443	0.559	0.681	0.804	0.929	1.051
0.890		0.212	0.385	0.592	0.820	1.059	1.299	1.538	1.771	1.996	2.212	2.418
0.913		0.609	0.972	1.361	1.756	2.143	2.515	2.869	3.203	3.518	3.811	4.085
0.918		0.771	1.196	1.641	2.085	2.515	2.924	3.309	3.670	4.006	4.320	4.612

**Fig 1:**
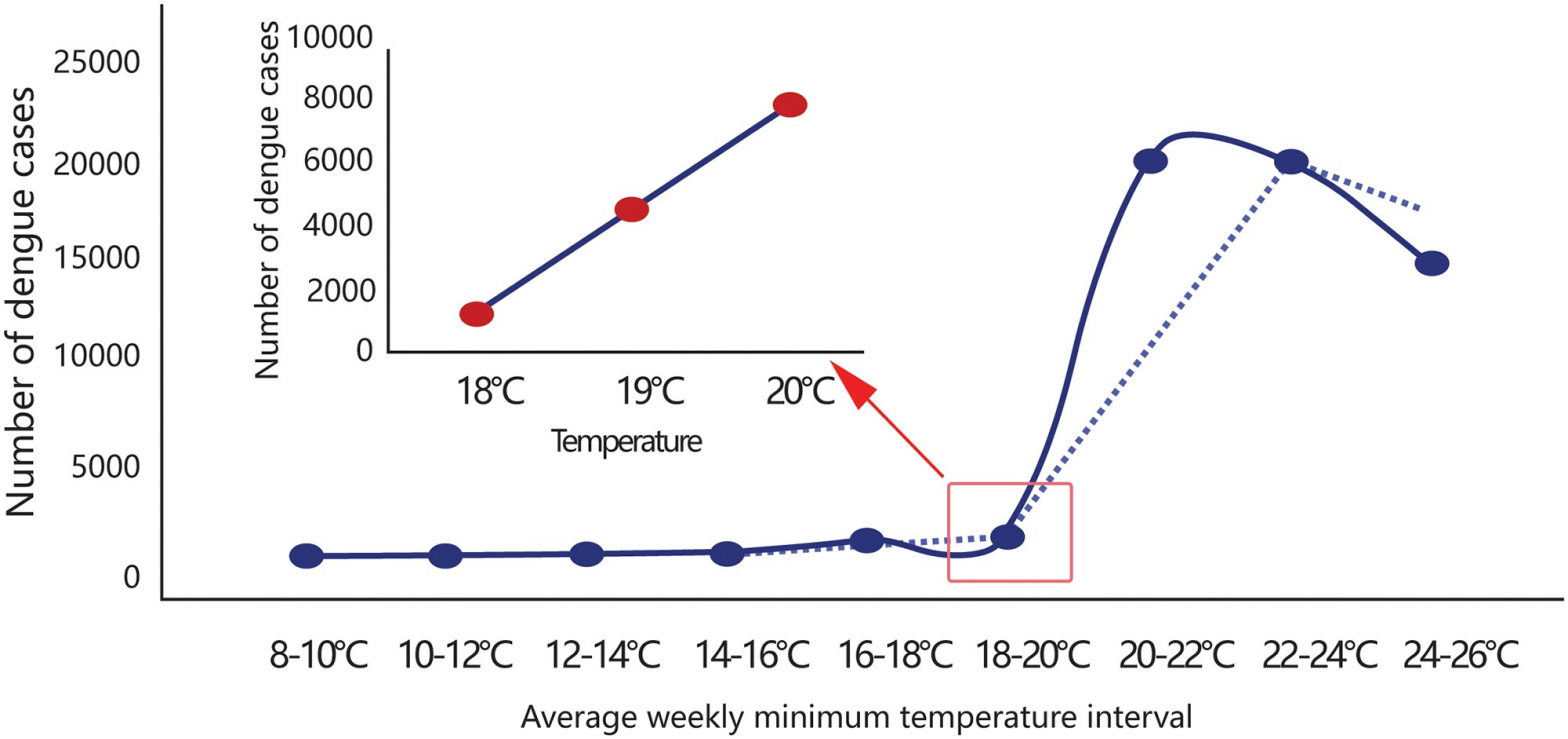
Minimum temperature range and cumulative number of cases. The blue solid line indicates the trends in dengue at 2°C intervals of minimum temperature from 8–26°C. The dotted blue line represents the moving average curve.

Based on the moving average equation of the minimum temperature interval and the cumulative number of cases, there was a temperature inflection point between the minimum temperature and number of cases at 18–20°C (Fig 1). When the minimum temperature was between 8–18°C, the number of cases only accounted for 2.01% of the total; when the minimum temperature was ≥18°C, the number of cases accounted for 97.97% of the total. We selected dengue fever cases with a minimum temperature ≥18°C for model construction.

### Correlation and interactive effects analysis of meteorological factors

Of the 477 weeks during the period between of 2008 to 2016, 282 weeks had minimum temperatures≥18°C, with the number of cases accounting for 97.91% of the total. Spearman rank correlation analysis showed that meteorological factors influencing dengue fever cases in these 282 weeks included mean temperature (r = 0.153, P = 0.010), maximum atmospheric pressure (r = 0.127, P = 0.033), minimum atmospheric pressure (r = 0.125, P = 0.036), mean atmospheric pressure (r = 0.124, P = 0.037), and mean relative humidity (r = −0.221, P <0.001)(Table 2). There was no correlation between rainfall and dengue based on the data from 2008 to 2014 in Guangdong, China. Regarding the lag between meteorological factors and dengue fever incidence, we constructed an ARIMA model to analyse the lag, but no lag was found (p = 0,q = 0).

**Table 2:** Spearman correlation coefficients between the number of dengue fever cases and meteorological factors, and among the meteorological factors when the minimum temperature was≥18°C.

Variable	Weekly mean temperature	Weekly maximum atmospheric pressure	Weekly minimum atmospheric pressure	Weekly mean atmospheric pressure	Weekly mean relative humidity	Weekly number of cases
Weekly mean temperature	1.000					
Weekly maximum atmospheric pressure	−0.663**	1.000				
Weekly minimum atmospheric pressure	−0.640**	0.987**	1.000			
Weekly mean atmospheric pressure	−0.657**	0.995**	0.993**	1.000		
Weekly mean relative humidity	−0.115	−0.256**	−0.246**	−0.251**	1.000	
Weekly number of cases	0.153*	0.127*	0.125*	0.124*	−0.221**	1.000

*Correlation was significant when significance level (two-tailed) was 0.05.

**Correlation was significant when significance level (two-tailed) was 0.01.

The results in Table 3 indicate that there were interactive effects between mean temperature and minimum atmospheric pressure and mean relative humidity (P = 0.05), minimum atmospheric pressure, and mean temperature (P = 0.039). The influence of meteorological factors on dengue fever is not a simple linear model. Taking temperature as an example, temperature will affect every stage of dengue transmission. Raising temperature at suitable temperature is beneficial to increase mosquito infection rate, transmission rate, and bite rate. But when the temperature is too high or too low, the infection rate, transmission rate and bite rate will decrease. This is because temperature, humidity, air pressure, rainfall, and other meteorological factors are interdependent and closely related, that is, there is an interaction between meteorological factors. The meteorological factors with interactive effects were further combined at different levels, and the analysis showed that when mean temperature was in the 25–28°C interval, minimum atmospheric pressure wasin the 997–1002 hPa interval, and mean relative humidity wasin the 73–81% interval, the intensity of the dengue epidemic was the greatest and had the highest number of cases (17,571 cases).

**Table 3:** Main effects and interactive effects of meteorological factors on dengue fever from the MANOVA.

Source	Type III sum of squares	Degrees of freedom	Mean square	F	P
Model	2640140798.805^a^	129	20466207.74	22.16	0
Maximum atmospheric pressure	1421935.067	4	355483.767	0.385	0.819
Minimum atmospheric pressure	2827359.382	4	706839.846	0.765	0.55
Mean atmospheric pressure	525878.693	4	131469.673	0.142	0.966
Mean temperature	399269.427	4	99817.357	0.108	0.98
Mean relative humidity	11322675.54	4	2830668.885	3.065	0.019
Maximum atmospheric pressure × Mean atmospheric pressure	2924365.503	8	365545.688	0.396	0.921
Maximum atmospheric pressure × Mean temperature	4997703.443	3	1665901.148	1.804	0.15
Maximum atmospheric pressure × Mean relative humidity	1345421.504	7	192203.072	0.208	0.983
Minimum atmospheric pressure × mean atmospheric pressure	763548.814	6	127258.136	0.138	0.991
Minimum atmospheric pressure × Mean temperature*	6132782.386	2	3066391.193	3.32	0.039
Minimum atmospheric pressure × Mean relative humidity	4608461.451	7	658351.636	0.713	0.661
Mean atmospheric pressure × Mean temperature	2276019.955	6	379336.659	0.411	0.871
Mean atmospheric pressure × Mean relative humidity	4936410.786	8	617051.348	0.668	0.719
Mean temperature × Mean relative humidity	714971.973	7	102138.853	0.111	0.998
Maximum atmospheric pressure × Mean atmospheric pressure × Mean temperature	93237.213	2	46618.606	0.05	0.951
Maximum atmospheric pressure × Mean atmospheric pressure × Mean relative humidity	5284841.301	8	660605.163	0.715	0.678
Maximum atmospheric pressure × Mean temperature × Mean relative humidity	4431357.149	2	2215678.575	2.399	0.095
Minimum atmospheric pressure × Mean atmospheric pressure × Mean temperature	1854.171	1	1854.171	0.002	0.964
Minimum atmospheric pressure × Mean atmospheric pressure × Mean relative humidity	509857.331	3	169952.444	0.184	0.907
Minimum atmospheric pressure × Mean temperature × Mean relative humidity*	3619832.058	1	3619832.058	3.919	0.05
Error	120062099.2	130	923554.609		
Total	2760202898	259			

^a^(R^2^ = 0.957 [adjusted R^2^ = 0.913]).

* Interactive effects were significant when significance level was≤0.05

### Prediction model of dengue

**Fig 2:**
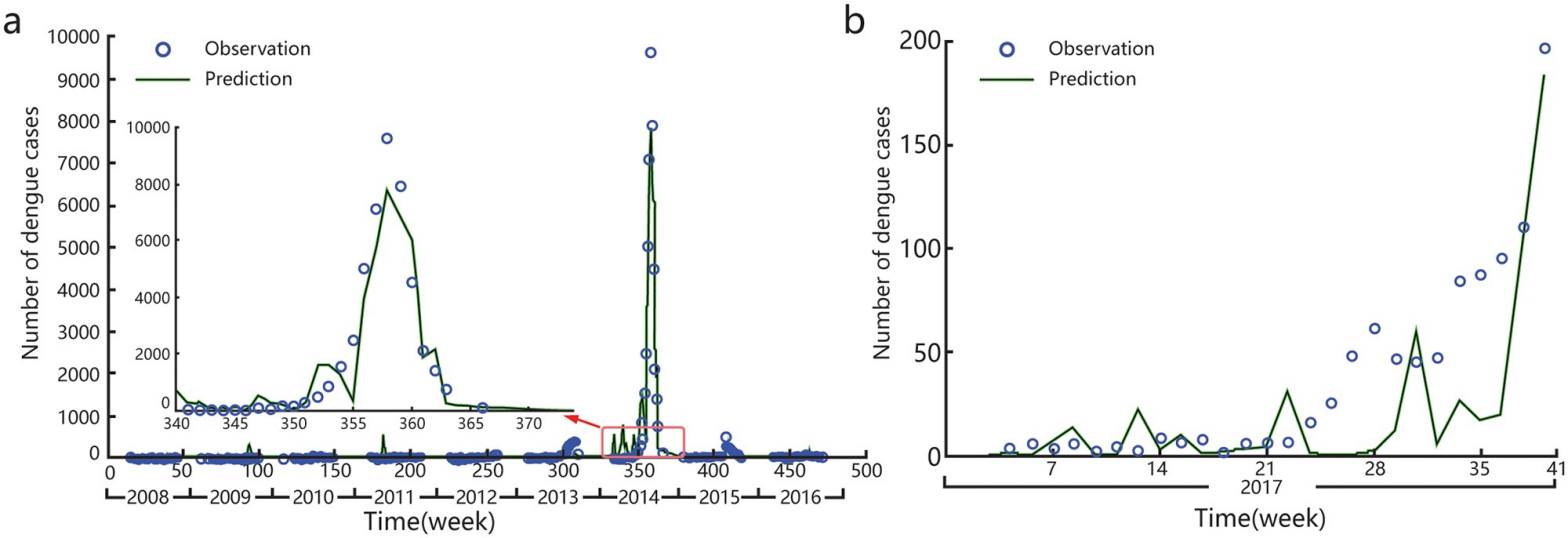
Model construction for dengue fever cases and meteorological factors. (a) Results of model fitting for a total of 477 weeks from 2008 to 2016. The green curve indicates logit fitting. (b) Results of model prediction for the first 41 weeks of 2017. The green curve indicates the predicted dengue cases.

Based on the above analysis, the key meteorological factors (mean temperature, mean relative humidity, mean atmospheric pressure, maximum atmospheric pressure, and minimum atmospheric pressure), interactive effect factors (mean temperature × minimum atmospheric pressure, mean temperature × minimum atmospheric pressure × mean relative humidity), mosquito-vector factor, and importation factor were used to constructan inverse cumulative normal distribution model, which was fitted to the number of cases in 2008–2016. The MLE was used to obtain the optimal parameter estimations: β_0_−β_9_ were 109.2567, 1.7889, −0.0588, −0.0369, 0.2211, −0.0778, −0.0018, 0.0000, 1.6671, and 0.8962, respectively. Moreover, α_1_, α_2_, and α_3_ were the model construction constants for the mosquito-vector factor, which were 1.6624, 27.2765, and 2.0651, respectively. β_0_ was the model construction constant, β_1_−β_9_ were the coefficients for the key meteorological factors, interactive effect factors, mosquito-vector factor, and importation factor. The simulation results showed good fit with the actual number of cases and a goodness of fit R^2^ = 0.72 excluding the year 2014 year (including 2014, R^2^ = 0.9248). Using this model, we predicted the number of dengue fever cases for the first 41weeks of 2017. The predicted values showed good fit with the observed values (goodness of fit R^2^ = 0.60, correlation coefficient = 0.8104)(Fig 2b). The onset of dengue fever is concentrated in the 38th to 42nd weeks of each year and is sporadic at other times, so the actual onset of dengue fever shows periodic outbreaks. Prediction models based on the onset of dengue fever also show intermittent outbreaks.

## Discussion

This study examined the relationship between dengue fever epidemics and meteorological factors based on weekly data from the past 9 years in Guangdong province. Our findings showed that an optimal minimum temperature was observed for dengue fever epidemics in Guangdong province. Analysis of data from 2008 to 2016 showed that the majority of dengue cases occurred when the minimum temperature was above 18°C. Studies have reported that when the minimum temperature is <18°C, virus propagation in the vector will be blocked; at <17°C, the mosquitoes will stop ingesting food; and at <16°C, there will be a prolonged mosquito larval stage[8,23–25]. Above the optimal minimum temperature, dengue fever cases accounted for nearly 98% of the total number of cases. Therefore, we constructed a prediction model for dengue fever that removed values below the optimal temperature to improve the accuracy and timeliness of dengue fever predictions.

In our study, the correlation coefficient between a single meteorological factor and the incidence of dengue fever is low, but when considering the interaction between meteorological factors, the correlation coefficient is high. It also shows that there is a real interaction between meteorological factors. Compared to single average temperature-related factors, the interactive effects led to slower growth in dengue epidemics, instead of sharp exponential growth when considering minimum air pressureandaverage relative humidity. This indicates that when studying the correlation between diseases and meteorological factors, using a single meteorological indicator will lead to poor accuracy. The interactive effects of different meteorological factors should be considered, which will provide a more valuable parameter of dengue fever model.

Chowellet et al[26]and Bambricket et al[27]used annual data to analyse factors influencing dengue epidemics in Peru and Australia, respectively. These studies found that the timing of dengue epidemics was closely correlated with seasonal temperature changes. In addition, Colon-Gonzalez et al[23]used monthly data to analyse the reasons for dengue outbreaks in Mexico and found that dengue epidemics were correlated with El Nino strength and minimum temperatures of the cool and dry seasons; no interactive effects were considered between different factors. However, using weekly data, we found that temperature, atmospheric pressure, and relative humidity had interactive effects on dengue epidemics. There are many factors that affect the incidence of dengue fever, including but not limited to mosquito factors, meteorological factors, and population mobility and so on. At a coarser scale (years), the overall change range of meteorological in same area is not obvious, so it is difficult to determine the relationship between the incidence and meteorological factors. The present results demonstrate that analysis based on weekly data can more precisely reveal the relationship between meteorological factors and dengue epidemics compared to that of monthly or yearly data. This implies that the scale of data analysis played a substantial role in these findings. Therefore, smaller scale data should be selected when analysing the patterns of dengue epidemics.

The majority of models for dengue epidemics using meteorological factors, mosquito-vector factor, preventive and control measures, and socioeconomic factors, such as seasonal time series models, regression analyses, and generalised additive models, are based on statistical models in quantitative research[7,9,28]. However, these do not consider the interactive effects among different factors or the occurrence of special events (e.g. imported cases, extreme climate). Furthermore, most studies cover a narrow range of time, a small number of cases, and fewer meteorological factors[8], leading to a poorer fit of the model. For example, the fitness of the SARIMA model constructed by Gharbietal. was 0.72[8], and that of the binomial regression model constructed by Adde et al. was 0.88[7]. Our study constructed a probitregression model, which incorporated interactive meteorological factors, the mosquito-vector factor, and the importation factor, based on dengue epidemic data from the last 9 years in Guangdong province. The model showed goodness of fit, at R^2^ = 0.72, indicating that the model calibration value fits the observed value effectively. Additionally, prediction of the actual number of cases for the first 41weeks of 2017 demonstrated that our constructed model has high reliability. Moreover, our model can predict the scale of dengue cases for the following week; hence its timeliness is higher than models constructed using months as the basic unit. Based on the above results, we can use meteorological forecasts to predict future dengue fever cases. Hence, our model can serve as a good theoretical basis for the prevention and control of dengue fever. A limitation of this study is that direct data on mosquito density were not obtained; instead, calculations were based on mean temperature. Moreover, this study was based on ecological data, thus factors besides meteorological factors, such as population growth and unreasonable waste disposal methods, should also be considered in future studies.

## Supporting information

S1 File Weekly meteorological and population data of Guangdong(2008–2016). (PDF)
