# Supplementary material for: Prediction model for dengue fever based on interactive effects between multiple meteorological factors in Guangdong, China (2008–2016)
Source: PLoS One. 2019 Dec 9;14(12):e0225811. doi: 10.1371/journal.pone.0225811 (PMC6901221; doi:10.1371/journal.pone.0225811)
Supplement: S1 File — (PDF) [file pone.0225811.s001.pdf]

Weekly Meteorologicaland population data of Guangdong(2008-2016)

Year	Population（ten thousand）	week	maximum temperature	minimum temperature	mean temperature	maximum pressure	minimum pressure	mean pressure	mean relative humidity	maximum wind speed	extreme wind speed
	9893	1	19.64710744	7.925619835	12.70743802	1013.063636	1007.993388	1010.466942	63.00826446	4.540495868	7.12892562
	9893	2	24.47816092	15.40172414	18.83678161	1006.73046	1001.579885	1004.268966	78.84482759	4.437356322	6.923563218
	9893	3	15.74685714	10.42171429	12.53028571	1013.165714	1007.715429	1010.594286	74.85714286	6.239428571	9.605142857
	9893	4	13.65485714	9.492	11.03714286	1011.888	1007.496	1009.610286	82.22285714	5.828571429	8.934285714
	9893	5	10.35895954	7.121965318	8.442196532	1010.714451	1005.730058	1008.110983	87.79190751	5.603468208	8.722543353
	9893	6	12.64152047	8.00994152	9.895906433	1011.940936	1007.161988	1009.500585	69.56725146	5.29122807	8.241520468
	9893	7	14.01085714	8.546285714	10.75428571	1014.954286	1010.426286	1012.703429	61.45714286	5.396	8.484571429
	9893	8	20.18457143	12.52628571	15.49657143	1013.038857	1008.274286	1010.740571	75.29142857	5.226285714	8.198857143
	9893	9	19.65170732	10.26341463	13.99365854	1010.83122	1004.661951	1008.02878	69.92195122	5.306829268	8.253170732
	9893	10	23.18831169	12.19376623	16.73090909	1007.934026	1002.904675	1005.475584	66.02857143	4.796103896	7.628831169
	9893	11	23.69873418	15.74936709	18.92721519	1006.957595	1001.868354	1004.526582	76.10126582	4.047468354	6.500632911
	9893	12	23.4952381	17.80079365	19.97142857	1004.250794	999.234127	1001.869841	77.71428571	4.596031746	7.288888889
	9893	13	24.55	15.975	19.4375	1004.196667	999.2766667	1001.92	76.95833333	3.858333333	6.319166667
	9893	14	21.98841463	16.56097561	18.82256098	1009.90061	1005.453049	1007.688415	84.90243902	3.907926829	6.450609756
	9893	15	28.33406593	22.55879121	24.84120879	1003.956593	999.3131868	1001.924176	84.7967033	4.946153846	8.245054945
	9893	16	28.19120879	21.57032967	24.3032967	1005.656593	1000.67033	1003.314286	74.72527473	5.16978022	8.628571429
	9893	17	25.32472527	19.27032967	21.62307692	1009.164835	1004.324725	1007.13022	73.29120879	4.183516484	6.724175824
	9893	18	27.6010989	21.35879121	23.85769231	1004.368132	1000.165385	1002.391758	73.58241758	3.737912088	6.131318681
	9893	19	28.50054945	21.98076923	24.54725275	1003.112637	998.6065934	1001.12967	74.21978022	4.761538462	7.880769231
	9893	20	29.90604396	20.47032967	24.65659341	1004.974176	1000.317582	1002.843407	80.81868132	4.213736264	6.85
	9893	21	27.93131868	22.33516484	24.48351648	1002.564286	998.7593407	1000.841758	64.00549451	4.213736264	6.99010989
	9893	22	29.78296703	24.0456044	26.13296703	1000.288462	996.3846154	998.4241758	90.95604396	4.917032967	8.667032967
	9893	23	29.20384615	23.16593407	25.38021978	1002.417033	999.0483516	1000.864835	78.28571429	4.543406593	7.725824176
	9893	24	30.00549451	24.45164835	26.60659341	1001.050549	997.0071429	999.071978	97.64835165	4.974175824	8.585164835
	9893	25	31.04230769	24.72637363	27.29725275	1000.445055	996.3263736	998.5835165	97.97802198	4.691208791	7.897802198
	9893	26	30.58791209	24.83681319	27.17307692	997.2769231	994	995.8505495	90.49450549	5.471428571	9.403296703
2008	9893	27	32.3521978	25.05934066	28.09725275	1003.088462	999.2296703	1001.342308	90.64285714	5.007692308	8.484065934
	9893	28	30.3043956	24.50989011	26.5510989	999.7637363	995.6142857	997.8494505	87.14835165	5.152747253	8.810989011
	9893	29	32.88846154	25.0510989	28.00879121	999.3615385	995.4214286	997.6620879	81.23076923	5.356043956	9.040659341
	9893	30	34.67362637	25.87692308	29.70384615	1000.256593	995.9065934	998.4098901	72.24725275	4.62967033	7.753846154
	9893	31	33.19065934	25.64065934	28.6032967	998.9016484	994.0181319	996.9005495	78.08241758	4.925274725	8.386263736
	9893	32	31.56483516	24.75054945	27.35769231	997.2703297	991.9291209	994.9307692	82.04945055	5.658241758	9.943406593
	9893	33	32.85384615	25.61098901	28.60879121	1000.974725	996.7164835	999.0802198	78.31868132	4.492307692	7.441208791
	9893	34	33.06483516	25.38076923	28.61593407	1000.971429	995.8131868	998.832967	75.65934066	5.424175824	9.343956044
	9893	35	33.20989011	25.50824176	28.70934066	1003.452198	999.5126374	1001.755495	77.00549451	4.252747253	6.889010989
	9893	36	31.81153846	24.36483516	26.96758242	1004.714286	1001.053297	1003.088462	81.77472527	4.831318681	8.097252747
	9893	37	33.73901099	24.95989011	28.5989011	1003.187363	998.2923077	1001.019231	72.47802198	4.41978022	7.280769231
	9893	38	34.18956044	25.41538462	28.98791209	1002.466484	998.267033	1000.564835	74.21978022	4.282417582	7.131318681
	9893	39	32.39725275	24.76483516	27.9	1001.50989	995.0879121	998.7291209	76.26923077	6.595604396	11.49175824
	9893	40	29.43571429	22.5978022	25.48791209	1005.775275	1001.447253	1003.76978	73.87912088	2.24456044	8.354395604
	9893	41	29.09725275	21.97032967	24.92582418	1009.314286	1005.236813	1007.33956	74.32417582	2.909725275	7.878021978
	9893	42	29.35989011	22.33296703	25.24120879	1009.548901	1005.70989	1007.634615	74.6043956	2.935989011	7.615384615
	9893	43	30.18296703	22.5543956	25.67582418	1010.298352	1005.930769	1008.203846	75.33516484	3.018296703	6.724725275
	9893	44	29.66978022	22.50714286	25.3489011	1010.462637	1006.220879	1008.350549	75.6043956	2.966978022	7.058241758
	9893	45	26.46758242	20.46043956	22.73681319	1010.391209	1006.191758	1008.36044	79.10989011	2.646758242	8.094505495
	9893	46	25.61373626	14.30769231	19.02472527	1013.131868	1007.622527	1010.410989	64.08241758	2.561373626	7.431318681
	9893	47	23.05384615	14.89175824	18.27307692	1015.866484	1010.530769	1013.476374	62.82417582	2.305384615	8.033516484
	9893	48	22.28406593	12.21153846	16.38186813	1018.415934	1013.049451	1015.869231	52.13736264	2.228406593	8.76043956
	9893	49	21.38241758	10.81538462	15.1967033	1017.298352	1010.981868	1014.37033	57.75824176	2.138241758	7.787362637
	9893	50	22.88571429	11.33626374	15.97802198	1014.27033	1008.858242	1011.726923	61.52747253	2.288571429	6.53956044
	9893	51	23.26538462	10.21923077	15.36538462	1015.314286	1010.147802	1012.848901	66.65384615	2.326538462	6.345054945
	9893	52	18.78076923	11.35274725	14.26483516	1016.554396	1011.037912	1013.998352	67.32967033	1.878076923	7.905494505
	9893	53	16.69320388	11.81262136	13.6961165	1016.341748	1012.18835	1014.095146	69.66019417	1.389708738	9.083495146

	10130	54	17.1474359	6.833333333	11.24358974	1020.058974	1014.553846	1017.34359	54.1025641	5.133333333	8.225641026
	10130	55	18.60384615	9.050549451	12.88956044	1018.187363	1012.873626	1015.698901	60.67582418	5.56043956	8.967582418
	10130	56	18.12472527	5.1	10.54285714	1022.051099	1015.651099	1019.237912	51.54945055	4.777472527	7.59010989
	10130	57	21.04725275	11.47252747	15.1478022	1014.048352	1007.674176	1011.041209	68.8021978	5.189010989	8.256593407
	10130	58	14.15824176	8.466483516	10.9032967	1014.097253	1009.548901	1011.714286	75.56043956	4.637912088	7.327472527
	10130	59	24.32087912	13.95769231	17.99065934	1013.154396	1008.163736	1010.616484	75.42307692	4.147252747	6.664835165
	10130	60	26.90714286	16.34725275	20.55549451	1007.770879	1002.425275	1005.233516	75.36813187	4.652197802	7.442857143
	10130	61	24.09835165	17.42637363	19.84230769	1010.496703	1004.385714	1007.619231	77.24175824	5.490659341	8.874725275
	10130	62	26.49120879	19.41538462	22.02637363	1007.334066	1002.603297	1004.975275	78.6978022	5.115384615	8.184065934
	10130	63	16.64120879	12.36483516	13.95604396	1011.582418	1006.007143	1008.822527	85.11538462	5.513736264	9.067032967
	10130	64	20.25659341	13.07087912	16.2043956	1012.356044	1006.407143	1009.324725	80.08241758	5.530769231	9.086813187
	10130	65	26.17967033	16.72087912	20.76263736	1008.294505	1002.988462	1005.746154	77.98901099	4.245604396	6.745604396
	10130	66	24.26483516	18.52637363	20.76813187	1007.79011	1003.034066	1005.473077	83.03296703	5.10989011	8.365934066
	10130	67	21.16318681	15.99285714	18.08846154	1012.418681	1007.871429	1010.319231	78.2967033	5.108791209	8.259340659
	10130	68	23.28681319	16.64725275	19.3478022	1010.429121	1005.930769	1008.183516	77.16483516	4.837362637	7.807692308
	10130	69	27.30274725	20.15549451	22.92637363	1003.008791	997.6313187	1000.416484	81.78021978	5.017582418	8.268131868
	10130	70	27.53846154	21.18901099	23.91373626	1002.359341	997.3236264	1000.073077	78.47802198	5.407692308	8.852747253
	10130	71	26.74505495	18.66593407	22.26043956	1009.769231	1005.592857	1007.869231	66.77472527	4.948351648	7.991208791
	10130	72	28.81098901	19.82527473	23.82527473	1006.42967	1001.780769	1004.274725	65.43956044	4.868131868	7.948901099
	10130	73	31.15879121	22.81923077	26.42197802	1005.042857	1000.636264	1003.019231	72.55494505	4.922527473	7.906043956
	10130	74	28.96868132	23.49230769	25.5989011	1003.887363	999.7445055	1001.93956	85.36263736	4.526923077	7.236813187
	10130	75	27.79505495	22.41813187	24.62802198	1002.996703	999.4472527	1001.381319	82.63186813	4.947802198	8.203846154
	10130	76	31.20824176	22.80659341	26.49450549	998.1291209	993.7016484	996.0247253	73.49450549	4.462087912	7.120879121
	10130	77	30.49010989	24.36153846	26.78351648	999.3225275	995.9159341	997.8197802	83.52197802	5.191208791	8.652747253
	10130	78	32.57307692	25.06318681	28.03681319	998.9862637	994.9681319	997.2214286	80.10989011	4.762087912	7.647802198
2009	10130	79	32.03186813	25.53186813	28.01538462	997.978022	993.9005495	996.2664835	82.52747253	5.691758242	9.506043956
	10130	80	32.3043956	25.47692308	28.26758242	999.8362637	996.2203297	998.7	79.70879121	2.152032967	9.797802198
	10130	81	32.9543956	25.57307692	28.58516484	999.4274725	995.017033	998	78.08791209	3.29543956	9.155494505
	10130	82	33.0989011	25.8010989	28.84010989	998.85	994.6263736	997.8	77.29120879	3.30989011	8.772527473
	10130	83	32.87527473	25.61098901	28.63791209	999.0269231	994.7604396	998.1	78.36813187	3.287527473	9.353296703
	10130	84	33.18076923	25.74340659	28.86703297	998.8489011	994.5934066	996.8	77.6978022	3.318076923	8.651098901
	10130	85	33.60494505	26.01868132	29.14725275	997.1043956	992.7505495	995.7	76.77472527	3.360494505	8.334615385
	10130	86	33.19175824	26.00879121	28.95604396	998.0956044	994.0582418	996.2	77.63186813	3.319175824	8.286813187
	10130	87	33.81428571	25.75714286	29.0043956	1000.621978	996.4291209	999.6	76.83516484	3.381428571	8.015934066
	10130	88	34.28626374	25.72142857	29.19175824	1000.555495	996.35	998.9	75.44505495	3.428626374	8.215384615
	10130	89	33.02637363	25.26373626	28.41043956	1001.867582	997.4758242	999	74.90659341	3.302637363	8.530769231
	10130	90	32.83021978	25.32472527	28.46978022	1002.08956	997.8263736	998.7	73.77472527	3.283021978	8.693956044
	10130	91	32.94175824	25.3543956	28.47032967	1002.084066	997.4	998.9	75.28571429	3.294175824	8.95989011
	10130	92	32.77692308	25.30659341	28.37362637	1002.443407	998.0049451	999.6	74.15384615	3.277692308	8.693956044
	10130	93	31.83021978	24.47912088	27.56153846	1003.844505	999.5813187	999.9	72.62637363	2.03456044	8.186813187
	10130	94	31.06428571	21.2521978	25.58021978	1004.342308	999.8307692	1002.117582	57.84065934	4.781318681	7.752197802
	10130	95	29.56043956	22.49230769	25.41263736	1008.519231	1003.963187	1006.251099	68.58241758	5.187362637	8.385164835
	10130	96	29.35384615	21.02252747	24.5978022	1006.993956	1002.570879	1004.887363	67.94505495	4.573076923	7.42032967
	10130	97	29.64395604	19.56703297	23.77857143	1010.285714	1005.51044	1007.826374	69.56043956	3.861538462	6.217032967
	10130	98	27.03846154	16.42582418	20.89725275	1015.152198	1009.355495	1012.487363	57.66483516	5.545054945	9.123626374
	10130	99	24.94285714	18.47637363	20.99010989	1006.806044	1001.923077	1004.48956	79.22527473	5.240659341	8.536813187
	10130	100	14.38846154	8.99010989	11.15384615	1017.747802	1012.493407	1015.437363	68.51098901	6.32967033	10.28736264
	10130	101	24.52142857	13.0543956	17.61923077	1013.028022	1007.826923	1010.489011	71.68131868	3.934615385	6.249450549
	10130	102	20.97692308	12.59175824	15.95164835	1016.333516	1011.245604	1013.820879	66.95054945	4.770879121	7.614835165
	10130	103	21.56813187	14.9	17.53736264	1011.718681	1007.223077	1009.419231	81.47252747	4.005494505	6.257692308
	10130	104	17.21483516	11.46593407	13.85	1017.226374	1012.780769	1014.926923	73.64835165	5.424175824	8.882417582
	10130	105	19.82087912	11.5956044	14.91263736	1015.396703	1010.139011	1012.880769	68.54945055	4.378571429	7.002197802
	10130	106	15.54	10.21692308	12.66461538	1012.472308	1007.116154	1009.77	80.26153846	5.320769231	8.700769231

	10441	107	16.14615385	12.88076923	14.25576923	1011.688462	1007.544231	1009.603846	85.76923077	4.051923077	6.503846154
	10441	108	16.38076923	11.04505495	13.18461538	1013.359341	1008.425275	1010.890659	83.65934066	4.671978022	7.529120879
	10441	109	16.86098901	9.443956044	12.4478022	1018.495055	1013.378022	1016.04011	69.36813187	4.90989011	7.81978022
	10441	110	20.71098901	12.77637363	15.92967033	1016.716484	1011.98956	1014.408242	82.06043956	4.531868132	7.273626374
	10441	111	19.45549451	13.68736264	15.90989011	1015.214286	1009.906593	1012.361538	82.98351648	4.441758242	7.089010989
	10441	112	21.57967033	16.3	18.28736264	1010.593956	1006.401099	1008.370879	84.80769231	4.243406593	6.727472527
	10441	113	21.86648352	15.27472527	17.68626374	1011.318681	1005.103297	1008.474725	83.16483516	5.664835165	9.204945055
	10441	114	10.98186813	6.491208791	8.434615385	1016.796154	1011.806044	1014.370879	81.98351648	4.925824176	8.064835165
	10441	115	24.78296703	17.07252747	20.20989011	1006.603846	1001.587912	1004.114286	83.24725275	4.568681319	7.37967033
	10441	116	27.50879121	20.67967033	23.37967033	1005.765934	1001.092857	1003.517033	81.00549451	5.596703297	9.145054945
	10441	117	16.55879121	9.845054945	12.55274725	1016.817582	1010.295055	1014.051099	74.84065934	5.193406593	8.376373626
	10441	118	24.49395604	16.65879121	19.78956044	1012.087912	1006.657143	1009.540659	80.0989011	4.482967033	7.148901099
	10441	119	24.37032967	16.11318681	19.4510989	1010.380769	1004.063736	1007.784066	70.38461538	5.634615385	9.217582418
	10441	120	23.61923077	16.22857143	19.31043956	1011.857692	1006.478022	1009.346154	74.76373626	5.32967033	8.705494505
	10441	121	22.11263736	17.39230769	19.42747253	1008.394505	1002.697253	1005.743956	88.09340659	4.367582418	7.026923077
	10441	122	22.78186813	16.32637363	18.92197802	1009.567582	1004.429121	1007.368681	85.38461538	5.203296703	8.443406593
	10441	123	25.66373626	18.92967033	21.73791209	1008.13022	1002.634615	1005.52967	81.59340659	5.238461538	8.614285714
	10441	124	25.1510989	18.3543956	21.12252747	1010.419231	1005.85	1008.255495	78.1043956	4.467032967	7.184615385
	10441	125	28.8978022	22.4021978	25.1489011	1003.007143	998.2302198	1000.726374	81.52197802	4.87032967	7.797802198
	10441	126	27.45824176	22.12967033	24.28351648	1003.493956	998.9730769	1001.425824	85.25824176	4.915384615	7.961538462
	10441	127	30.58956044	24.11758242	26.80989011	1002.770879	998.332967	1000.741758	83.02747253	5.538461538	9.259340659
	10441	128	30.65824176	22.38351648	25.92362637	1001.07967	996.9131868	999.2489011	75.41208791	4.625274725	7.553846154
	10441	129	27.82472527	21.89615385	24.24285714	1002.587363	999.2994505	1001.067582	82.00549451	4.172527473	6.764835165
	10441	130	28.78791209	23.16043956	25.46813187	1002.800549	999.2725275	1001.121429	82.23626374	4.473076923	7.128021978
	10441	131	31.15659341	25.72252747	27.86538462	1000.304396	997.1565934	998.7912088	85.88461538	5.496153846	9.210989011
	10441	132	30.75769231	25.46483516	27.42307692	999.6318681	995.789011	997.9021978	85.64285714	5.482417582	8.915934066
2010	10441	133	32.28681319	25.52472527	16.5532967	1001.284615	997.95	999.7620879	80.99450549	4.707692308	7.725274725
	10441	134	34.71098901	28.49450549	27.40604396	1000.870879	997.1895604	999.2461538	72.32967033	5.581868132	9.111538462
	10441	135	33.71483516	23.97252747	26.24395604	1001.940659	998.6098901	1000.556044	74.5	5.717582418	9.33956044
	10441	136	32.54285714	22.67582418	25.14285714	1002.498901	998.332967	1000.594505	79.63186813	5.582967033	9.326923077
	10441	137	32.32802198	19.17032967	25.43351648	1001.165385	997.7247253	999.5945055	83.01098901	5.194505495	8.537362637
	10441	138	34.15	18.73626374	25.47582418	1001.568132	996.821978	999.4049451	75.3956044	5.482967033	8.958791209
	10441	139	33.92032967	19.86813187	26.24285714	1000.604396	997.0637363	999.0307692	76.97802198	5.209340659	8.406043956
	10441	140	32.90164835	19.33516484	25.12637363	1005.050549	1001.107692	1003.291209	78.79120879	5.356593407	8.692857143
	10441	141	32.54340659	19.52197802	25.02527473	1002.675824	998.8340659	1001.103846	79.86813187	5.547252747	9.035164835
	10441	142	32.13736264	20.6043956	25.19450549	998.9467033	995.0082418	997.1576923	76.80769231	4.811538462	7.925824176
	10441	143	33.04945055	17.11538462	25.24285714	1000.992308	996.7961538	999.2307692	81.36263736	4.777472527	7.758241758
	10441	144	32.53846154	16.32417582	24.41703297	1004.370879	1000.466484	1002.627473	81.23076923	4.654395604	7.432967033
	10441	145	30.56208791	20.25274725	23.90824176	1003.232967	997.8697802	1000.818132	84.16483516	5.195054945	8.637362637
	10441	146	31.09450549	9.014835165	24.18021978	1007.622527	1003.607692	1005.747253	78.98901099	4.492857143	7.174175824
	10441	147	27.13241758	21.68736264	23.9478022	1007.304945	1003.596703	1005.404945	71.84065934	4.784065934	7.92967033
	10441	148	29.26923077	23.12912088	25.53351648	1006.516484	1002.433516	1004.637363	81.28021978	4.469230769	7.189010989
	10441	149	27.5967033	21.27362637	24.01703297	1004.563187	999.8142857	1002.253846	67.05494505	5.866483516	9.677472527
	10441	150	23.61153846	16.34230769	19.43461538	1012.347802	1007.532967	1010.214286	61.03846154	5.800549451	9.573076923
	10441	151	23.81153846	14.09835165	18.29340659	1014.498352	1009.532418	1011.984066	65.95054945	4.615384615	7.663736264
	10441	152	26.99505495	14.56208791	19.67252747	1012.528571	1007.851648	1010.213736	64.0989011	4.552197802	7.321978022
	10441	153	24.60714286	16.3010989	19.68296703	1013	1008.108791	1010.68022	71.82417582	4.568131868	7.377472527
	10441	154	23.78076923	15.88956044	19.1467033	1010.193407	1005.935714	1008.167582	70.54945055	4.326923077	7.028571429
	10441	155	24.37087912	15.48406593	19.1	1012.155495	1007.338462	1009.803297	71.25824176	4.053846154	6.523626374
	10441	156	22.11318681	13.26813187	16.87527473	1010.843407	1005.592857	1008.32967	63.13186813	4.578571429	7.422527473
	10441	157	18.01758242	9.765934066	13.16153846	1011.497253	1005.745604	1008.765934	78.04395604	5.227472527	8.514285714
	10441	158	21.74450549	12.33846154	16.0478022	1010.996154	1006.031868	1008.50989	73.13186813	4.087362637	6.525824176
	10441	159	19.45	7.231410256	12.11346154	1014.619872	1008.783333	1011.833333	60.45512821	4.563461538	7.255128205

	10505	160	17.56538462	8.073076923	11.98461538	1013.938462	1009.026923	1011.526923	49.5	5.438461538	8.757692308
	10505	161	12.1467033	6.987912088	9.083516484	1017.045055	1011.559341	1014.432967	70.58241758	5.515384615	8.836813187
	10505	162	13.40604396	7.265384615	9.681868132	1015.483516	1009.989011	1012.952198	64.76373626	5.730769231	9.1
	10505	163	14.46428571	7.051098901	10.07197802	1016.552198	1011.29011	1014.003297	59.63186813	5.60989011	8.995604396
	10505	164	14.56098901	8.571978022	10.79725275	1019.673077	1014.588462	1017.328022	67.16483516	5.531318681	8.824725275
	10505	165	18.72967033	8.713186813	12.68131868	1016.757692	1010.846703	1014.021978	61.56043956	4.703846154	7.42032967
	10505	166	22.36813187	12.58901099	16.41043956	1008.721429	1002.76044	1005.909341	71.44505495	5.275274725	8.426373626
	10505	167	12.03076923	8.52032967	9.908241758	1013.54011	1008.829121	1011.082967	85.72527473	5.13021978	8.212087912
	10505	168	21.15769231	13.83571429	16.6543956	1012.106044	1007.385165	1009.635714	75.71428571	4.266483516	6.726373626
	10505	169	22.49450549	14.12032967	17.25384615	1011.865934	1006.206593	1009.301099	70.33516484	5.464835165	8.61978022
	10505	170	18.05	13.07252747	15.04725275	1014.01044	1008.735714	1011.668132	77.05494505	4.973626374	7.933516484
	10505	171	19.92637363	12.47582418	15.50879121	1013.884066	1007.252747	1010.518132	73.06593407	5.728571429	9.07032967
	10505	172	20.14945055	13.81483516	16.20054945	1013.681868	1007.912637	1011.291758	72.44505495	5.386263736	8.46043956
	10505	173	22.81318681	12.98461538	17.23736264	1014.645055	1008.96978	1011.971978	60.10989011	4.72967033	7.543406593
	10505	174	24.98076923	16.71318681	19.91648352	1011.917033	1006.396703	1009.385714	70.42857143	4.883516484	7.685164835
	10505	175	28.7543956	19.85274725	23.59340659	1008.845055	1003.024725	1006.07967	70.86263736	4.808791209	7.512087912
	10505	176	27.71373626	19.32142857	22.78571429	1007.091209	1002.407143	1004.988462	70.11538462	5.418131868	8.817032967
	10505	177	29.31483516	20.13791209	24.20879121	1005.052198	1000.35	1002.792308	71.46153846	4.455494505	6.934065934
	10505	178	27.09010989	21.57472527	23.66318681	1003.803297	999.5725275	1001.869231	87.50549451	4.663736264	7.358791209
	10505	179	29.94725275	23.63846154	26.13791209	1001.983516	997.9505495	1000.204396	82.42307692	5.17967033	8.358241758
	10505	180	29.21648352	21.41098901	24.73846154	1004.634066	1000.342308	1002.626923	78.38461538	4.407142857	7.018681319
	10505	181	27.85054945	20.67637363	23.74120879	1002.648352	998.5247253	1000.86044	72.32417582	4.812087912	7.728571429
	10505	182	32.33241758	23.1510989	27.22582418	1002.976374	998.2467033	1000.931319	70.03846154	4.631868132	7.3
	10505	183	33.20879121	26.01813187	28.78956044	1000.034615	995.8950549	998.2247253	77.57692308	5.844505495	9.445054945
	10505	184	31.75989011	25.54615385	28.02802198	1000.024176	996.2796703	998.2395604	81.67032967	5.677472527	9.341758242
	10505	185	32.69450549	25.42527473	28.44450549	995.5307692	991.1961538	993.5576923	78.23626374	5.687912088	9.399450549
2011	10505	186	31.36153846	25.04395604	27.44395604	999.032967	995.7423077	997.5758242	83.93406593	5.675274725	9.235164835
	10505	187	34.05	26.21868132	29.5510989	999.5241758	995.4236264	997.7192308	74.15934066	5.082967033	7.936263736
	10505	188	31.46428571	25.30879121	27.44450549	996.0478022	992.5423077	994.5137363	85.27472527	5.015384615	8.053846154
	10505	189	31.61758242	25.20549451	27.71208791	996.1934066	992.9532967	994.7126374	83.96703297	4.89010989	7.773626374
	10505	190	34.0543956	25.78351648	29.28241758	999.8653846	995.4197802	997.9043956	74.6043956	5.522527473	8.979120879
	10505	191	34.10494505	25.54615385	29.14120879	997.8016484	993.5324176	995.989011	75.16483516	4.516483516	7.143956044
	10505	192	32.57692308	25.70549451	28.30989011	999.2989011	995.9736264	997.8252747	81.17582418	5.27032967	8.335164835
	10505	193	33.74230769	25.33186813	28.94285714	1003.941209	1000.332418	1002.308791	73.34065934	5.148901099	8.302197802
	10505	194	33.78516484	25.28241758	28.78186813	1001.067582	997.006044	999.3285714	73.61538462	4.893406593	7.767582418
	10505	195	32.5956044	25.5978022	28.35934066	996.656044	992.8065934	994.8758242	75.03846154	4.750549451	7.642857143
	10505	196	33.20384615	25.38351648	28.46538462	1002.143956	998.2461538	1000.443956	76.95604396	4.656043956	7.470879121
	10505	197	32.96153846	24.65164835	27.87307692	1002.184615	997.9192308	1000.297802	76.82967033	5.285714286	8.636813187
	10505	198	29.26538462	22.1478022	25.16978022	1004.995055	1000.778022	1003.027473	67.03296703	5.644505495	9.106593407
	10505	199	30.3532967	23.76263736	26.56648352	1002.293407	997.106044	1000.001099	74.79120879	6.37967033	10.71483516
	10505	200	25.73241758	20.4010989	22.61483516	1007.563187	1004.255495	1005.902747	76.81318681	5.806043956	9.546703297
	10505	201	28.42857143	22.62527473	24.78956044	1006.634066	1002.506044	1004.665385	81.94505495	4.697802198	7.52967033
	10505	202	28.49340659	18.62417582	22.80824176	1010.007692	1005.600549	1007.782418	67.74725275	4.32967033	6.971428571
	10505	203	26.7967033	19.86483516	22.63296703	1011.059341	1006.758242	1008.931319	73.2967033	4.609340659	7.268131868
	10505	204	28.4456044	20.07472527	23.57307692	1009.606593	1005.367582	1007.488462	73.10989011	4.380769231	6.923626374
	10505	205	24.62032967	18.36923077	20.77197802	1007.725824	1003.65989	1005.633516	78.47802198	5.514285714	8.985164835
	10505	206	26.83516484	19.32032967	22.28846154	1009.949451	1004.836264	1007.390659	79.12087912	4.245604396	6.867582418
	10505	207	23.8956044	16.84615385	19.73516484	1015.397253	1010.456593	1013.062637	66.58791209	4.781318681	7.742857143
	10505	208	23.37857143	13.27857143	17.18901099	1011.514835	1006.142857	1009.062637	71.09340659	5.053846154	8.03956044
	10505	209	19.6978022	12.53516484	15.55549451	1015.302747	1010.354396	1012.967582	63.5989011	5.658791209	9.11043956
	10505	210	19.01153846	10.05494505	13.78406593	1017.140659	1011.875824	1014.62033	51.65934066	5.28956044	8.401648352
	10505	211	18.80989011	10.66373626	13.96428571	1016.713736	1011.427473	1014.170879	57.08791209	5.223076923	8.467032967
	10505	212	19.00604396	10.38461538	13.90659341	1018.493407	1013.303846	1015.987363	62.63186813	4.698351648	7.517582418

	10594	213	14.15054945	9.218131868	11.13626374	1017.887912	1012.810989	1015.23956	76.25274725	5.063186813	8.223076923
	10594	214	16.33791209	10.76538462	12.98131868	1016.603846	1011.687912	1014.071429	79.41208791	4.277472527	6.897252747
	10594	215	17.35164835	12.38901099	14.28626374	1008.402198	1003.834615	1005.886813	86.05494505	4.536263736	7.195054945
	10594	216	10.94175824	6.602197802	8.352197802	1013.653846	1008.847802	1011.377473	86.32967033	5.04010989	8.081318681
	10594	217	15.95054945	10.3978022	12.53681319	1015.212637	1010.138462	1012.672527	75.39010989	4.773076923	7.571978022
	10594	218	15.81758242	10.24175824	12.46263736	1012.491758	1006.302747	1009.85989	79.58241758	5.384615385	8.508791209
	10594	219	19.87362637	12.32582418	15.13846154	1012.202747	1006.720879	1009.843956	76.23076923	4.858791209	7.663186813
	10594	220	20.82637363	13.98076923	16.74395604	1007.76978	1002.192308	1005.18022	79.46703297	4.650549451	7.321428571
	10594	221	15.61648352	10.82417582	12.75934066	1008.937912	1003.898901	1006.515385	88.51098901	4.745054945	7.621428571
	10594	222	19.21318681	14.57472527	16.37252747	1005.839011	1000.826923	1003.486264	88.92307692	5.091758242	8.180769231
	10594	223	18.66868132	13.49175824	15.59615385	1011.443407	1007.018681	1009.176923	87.92307692	4.076923077	6.544505495
	10594	224	24.91813187	17.18296703	20.25549451	1008.993407	1003.428022	1006.306044	78.74725275	5.831868132	9.361538462
	10594	225	25.47307692	15.72747253	19.9510989	1012.953846	1007.456593	1010.539011	63.6043956	5.179120879	8.355494505
	10594	226	24.84835165	18.47307692	21.09340659	1010.173077	1005.057692	1007.738462	77.34065934	5.134615385	8.239010989
	10594	227	27.46813187	20.83296703	23.43571429	1005.882967	1001.026374	1003.603846	85.26373626	4.993956044	7.796153846
	10594	228	26.1456044	21.09835165	23.01758242	1002.529121	998.1021978	1000.345604	87.53296703	5.541758242	8.82967033
	10594	229	27.9032967	21.08736264	24.01923077	1002.018681	996.8587912	999.6318681	82.45054945	6.07967033	9.884615385
	10594	230	31.17252747	24.37967033	27.02472527	999.7467033	995.0923077	997.5868132	84.01648352	5.745604396	9.307692308
	10594	231	31.51868132	24.13241758	27.0978022	1001.776374	997.5049451	999.9126374	79.74725275	5.207692308	8.176373626
	10594	232	30.34505495	23.9010989	26.39340659	1000.32033	996.2247253	998.3807692	84.38461538	5.045054945	8.187362637
	10594	233	30.32087912	23.67252747	26.42032967	1000.014286	996.4543956	998.428022	77.84615385	4.911538462	7.895604396
	10594	234	29.18461538	23.95274725	25.94120879	1001.199451	997.7071429	999.5703297	83.81318681	4.663736264	7.476923077
	10594	235	31.84285714	25.09175824	27.88516484	997.2692308	993.4126374	995.5802198	79.57692308	4.77967033	7.500549451
	10594	236	31.57802198	25.25659341	27.72747253	993.6708791	990.0675824	991.9675824	82.62087912	5.633516484	9.176373626
	10594	237	30.8956044	25.12802198	27.2978022	994.4126374	990.156044	992.3368132	85.56593407	5.506593407	9.134065934
	10594	238	31.72582418	25.61153846	28.07362637	996.6285714	993.0467033	995.0230769	81.5	5.751648352	9.41978022
2012	10594	239	32.18296703	24.94120879	28.05549451	999.139011	995.7675824	997.6417582	79.52197802	5.306043956	8.558791209
	10594	240	33.58351648	26.16923077	29.39725275	999.0824176	995.3928571	997.4478022	74.5989011	5.507692308	8.722527473
	10594	241	34.01428571	26.28461538	29.3467033	998.7	994.4928571	996.910989	77.91208791	5.450549451	8.652197802
	10594	242	30.27087912	24.76758242	26.79175824	996.1587912	990.8818681	993.6076923	86.18681319	6.087362637	10.17472527
	10594	243	34.01373626	25.3467033	28.93351648	993.4	989.1741758	991.4472527	76.45604396	4.554945055	7.392307692
	10594	244	33.14725275	25.09340659	28.2521978	994.8423077	991.3153846	993.2840659	80.76923077	5.138461538	8.288461538
	10594	245	32.46648352	25.34450549	28.07197802	997.5472527	993.1934066	995.7351648	83.09340659	5.677472527	9.260989011
	10594	246	32.59395604	25.00164835	27.91703297	999.7532967	995.9137363	997.9945055	80.02197802	5.073626374	8.02967033
	10594	247	33.58406593	25.29120879	28.64615385	998.8186813	994.8791209	997.0214286	76.53846154	5.206043956	8.35
	10594	248	31.93351648	24.44505495	27.38406593	1004.490659	1001.024725	1002.936264	81.15934066	5.242857143	8.368131868
	10594	249	31.57747253	23.83296703	26.96153846	1003.682967	999.482967	1001.877473	74.95604396	5.280769231	8.319230769
	10594	250	30.11758242	22.27967033	25.66373626	1004.474176	1000.646703	1002.646154	70.84065934	4.153846154	6.591208791
	10594	251	30.72472527	23.69285714	26.6021978	1003.175824	999.5098901	1001.547802	72.99450549	5.117032967	8.291208791
	10594	252	29.8543956	20.01978022	24.26483516	1006.767582	1002.45989	1004.754396	65.76923077	4.393406593	7.007142857
	10594	253	29.78901099	20.78516484	24.60934066	1007.542308	1002.867582	1005.295055	65.83516484	4.369230769	7.004945055
	10594	254	29.27307692	20.22802198	24.01813187	1009.332967	1004.72033	1007.062637	70.61538462	4.591208791	7.28956044
	10594	255	29.52527473	20.47252747	24.2021978	1009.06978	1004.59011	1006.828022	74.32417582	4.475824176	7.155494505
	10594	256	26.03901099	18.1010989	21.41208791	1009.48956	1004.897253	1007.229121	75.43406593	5.168681319	8.447252747
	10594	257	26.13296703	19.48791209	22.17032967	1008.516484	1004.06978	1006.343407	73.08791209	4.894505495	7.816483516
	10594	258	24.52032967	17.32747253	20.13956044	1011.281868	1017.581868	1009.007143	77.86813187	5.242307692	8.409340659
	10594	259	23.38626374	17.52802198	19.92417582	1009.167582	1003.939011	1006.678571	82.88461538	4.583516484	7.429120879
	10594	260	18.81263736	14.40934066	16.33791209	1009.945604	1004.927473	1007.470879	92.56043956	4.692307692	7.53021978
	10594	261	18.63076923	12.86758242	15.16483516	1011.817033	1006.795055	1009.39011	78.73076923	4.933516484	7.924175824
	10594	262	21.46538462	14.34835165	17.20879121	1011.823626	1007.309341	1009.553846	78.41758242	4.652747253	7.362087912
	10594	263	20.02967033	13.69945055	16.14285714	1012.564286	1006.962637	1010.071978	82.07142857	5.35	8.558241758
	10594	264	17.60604396	10.2543956	13.39010989	1014.96044	1009.281868	1012.09011	75.26373626	5.776923077	9.231318681
	10594	265	12.69807692	4.434615385	7.598076923	1018.1	1011.65	1015.380769	50.5	7.826923077	12.78461538

	10644	266	14.06153846	7.958461538	10.56	1015.343846	1010.226923	1012.868462	69.20769231	5.03	7.861538462
	10644	267	15.47912088	9.136813187	11.60879121	1014.267582	1009.308791	1011.800549	70.52747253	5.063736264	7.926923077
	10644	268	19.07197802	10.05659341	13.59175824	1015.775824	1010.043407	1013.219231	70.57692308	4.908241758	7.682967033
	10644	269	21.20989011	13.87472527	16.67582418	1013.288462	1008.53956	1010.775824	78.05494505	4.577472527	7.036813187
	10644	270	23.06373626	13.35	17.22747253	1014.72033	1010.265385	1012.371978	74.02197802	4.647252747	7.244505495
	10644	271	22.12472527	15.80714286	18.09945055	1011.558242	1006.311538	1009.232418	83.62087912	5.37032967	8.462637363
	10644	272	19.36098901	12.37197802	15.05824176	1013.257143	1008.032967	1010.782967	77.64835165	5.012637363	7.896703297
	10644	273	20.80714286	13.43791209	16.22747253	1012.508242	1007.303297	1010.001099	76.92857143	5.419230769	8.625824176
	10644	274	23.67307692	16.6543956	19.42417582	1010.334066	1004.63022	1007.867033	82.87362637	4.881318681	7.684065934
	10644	275	23.82142857	11.5467033	16.73901099	1014.335165	1008.650549	1011.646154	65.89010989	4.691208791	7.312637363
	10644	276	25.24340659	16.79835165	20.16483516	1010.614286	1005.198352	1008.016484	75.99450549	5.154945055	8.110989011
	10644	277	26.31648352	19.77362637	22.39065934	1005.456593	1000.165934	1002.77033	84.47802198	5.362637363	8.430769231
	10644	278	22.72307692	18.13571429	19.91153846	1005.771429	1000.535165	1003.221429	88.33516484	6.104395604	9.990659341
	10644	279	23.41098901	18.5521978	20.40549451	1004.580769	998.510989	1001.760989	88.91758242	5.413736264	8.710989011
	10644	280	19.9989011	14.73626374	17.06153846	1010.02033	1005.153846	1007.590659	80.44505495	4.673626374	7.457142857
	10644	281	28.04285714	20.19340659	23.32307692	1002.962088	998.1395604	1000.702198	84.63186813	4.756043956	7.412087912
	10644	282	25.25	19.6956044	21.87197802	1006.671429	1001.991209	1004.548901	85.98901099	5.185164835	8.185714286
	10644	283	24.99230769	19.73241758	21.88021978	1004.135714	1000.284615	1002.346703	84.76373626	5.242857143	8.543406593
	10644	284	27.62802198	21.81593407	24.05604396	1003.854396	999.5423077	1001.791209	87.95604396	4.766483516	7.607692308
	10644	285	30.1967033	23.78076923	26.41648352	999.3956044	995.1368132	997.5159341	86.5989011	5.030769231	8.021428571
	10644	286	30.51104972	23.89447514	26.3467033	999.7791209	995.721978	997.9917582	87.91758242	5.724861878	9.499447514
	10644	287	32.57527473	24.9467033	28.1532967	1001.534615	997.260989	999.5950549	82.17032967	5.993956044	9.47967033
	10644	288	32.23846154	24.87252747	27.71043956	1000.765934	996.4714286	998.7796703	82.25824176	5.891758242	9.415934066
	10644	289	28.78626374	23.23461538	25.48351648	997.321978	994.1093407	995.9043956	85.52197802	5.35989011	8.759340659
	10644	290	33.17747253	25.59395604	28.71648352	996.5274725	992.3296703	994.5901099	80.85714286	5.075824176	8.11978022
	10644	291	32.26043956	26.05824176	28.51593407	998.0401099	994.7593407	996.660989	81.71428571	6.279120879	10.31373626
2013	10644	292	32.87307692	25.61263736	28.82582418	1000.295055	996.3824176	998.678022	77	6.02967033	9.712087912
	10644	293	33.26923077	25.03241758	28.4521978	1000.472527	996.0796703	998.4714286	77.71428571	5.792857143	9.128021978
	10644	294	31.18296703	24.78296703	27.02967033	999.5692308	995.3521978	997.7587912	85.46153846	5.815934066	9.462637363
	10644	295	30.92582418	24.73131868	27.02362637	999.4659341	996.1	997.8071429	86.13736264	5.68021978	9.36978022
	10644	296	32.35549451	24.76758242	27.92252747	999.5895604	995.4016484	997.7593407	81.91208791	5.885164835	9.758791209
	10644	297	33.75934066	25.26208791	29.05	1002.625824	998.6917582	1000.83022	77.14285714	5.118681319	8.171978022
	10644	298	30.5489011	24.72362637	27.18461538	996.7450549	991.6247253	994.2758242	84.87362637	6.476923077	10.77967033
	10644	299	31.3021978	25.11648352	27.4532967	993.5335165	989.021978	991.467033	87.54395604	5.147802198	8.290659341
	10644	300	33.01263736	25.18021978	28.26868132	999.0681319	995.1873626	997.4549451	82.93406593	4.784065934	7.547252747
	10644	301	29.71098901	23.69010989	25.99340659	1003.726374	1000.287912	1002.142308	85.69230769	4.860989011	7.829120879
	10644	302	33.04395604	24.64395604	27.89285714	1004.328022	999.7456044	1002.294505	79.68131868	4.884615385	7.78021978
	10644	303	33.03681319	24.15549451	28.03296703	1000.269231	995.5769231	998.0142857	72.28571429	5.613186813	9.196703297
	10644	304	29.95054945	23.54725275	26.16538462	1000.693407	993.8928571	998.0412088	77.73626374	6.73956044	10.87417582
	10644	305	29.54285714	21.53461538	25.00549451	1005.246703	1001.184615	1003.27033	71.38461538	4.983516484	8.108241758
	10644	306	30.60494505	22.17362637	25.79450549	1004.031868	999.9994505	1002.081868	69.51098901	5.116483516	8.181318681
	10644	307	28.06978022	21.33351648	24.07362637	1009.276374	1005.128022	1007.284066	68.08241758	5.375824176	8.596153846
	10644	308	27.26043956	18.05164835	22.1010989	1009.968132	1005.225824	1007.668132	57.54395604	5.356593407	8.634615385
	10644	309	27.77692308	17.94450549	22.17637363	1010.70989	1005.976374	1008.351934	68.7032967	4.407692308	7.023626374
	10644	310	27.16208791	20.02307692	22.92417582	1010.908791	1006.485165	1008.723626	75.5	5.103846154	8.274175824
	10644	311	23.43626374	17.96263736	20.12142857	1009.970879	1005.582418	1007.803846	80.68681319	5.792857143	9.474175824
	10644	312	23.73736264	15.34120879	18.87747253	1013.05989	1008.508791	1010.714835	64.21428571	4.411538462	7.056043956
	10644	313	20.8043956	12.2532967	15.72362637	1013.62967	1008.108242	1011.137912	65.10989011	5.700549451	9.267032967
	10644	314	22.41593407	9.678571429	14.97472527	1013.050549	1008.121978	1010.608791	61.84615385	4.249450549	6.725824176
	10644	315	19.61153846	13.28956044	15.95	1010.785714	1005.90989	1008.41978	70.18681319	4.904945055	7.986813187
	10644	316	13.69175824	8.577472527	10.63626374	1014.562088	1010.123077	1012.23022	76.35164835	6.139010989	9.878571429
	10644	317	16.28076923	6.992307692	10.90384615	1017.285165	1011.91044	1014.905495	52.61538462	5.225274725	8.348901099
	10644	318	17.31410256	4.942307692	9.979487179	1016.823077	1010.930769	1013.920513	54.23076923	3.816666667	5.930769231

	10724	319	21.64903846	9.534615385	14.50865385	1011.564423	1006.442308	1008.975	66.28846154	3.805769231	5.860576923
	10724	320	19.12692308	11.30054945	14.41043956	1014.096154	1008.97967	1011.664835	71.58241758	5.041208791	7.987912088
	10724	321	17.70384615	8.06043956	11.94725275	1018.491758	1013.692308	1016.208242	65.22527473	5.502747253	8.811538462
	10724	322	19.21813187	6.890659341	12.12967033	1016.802747	1011.531319	1014.220879	58.71978022	4.941208791	7.830769231
	10724	323	23.93131868	12.52252747	17.05934066	1012.638462	1007.681319	1010.018681	74.15934066	4.180769231	6.59010989
	10724	324	22.50604396	13.91758242	17.26022099	1005.468681	1000.45	1002.98011	80.24861878	4.965384615	7.771978022
	10724	325	9.391758242	5.593956044	7.15989011	1013.987363	1008.75989	1011.683516	75.8956044	5.832417582	9.535164835
	10724	326	16.44615385	8.033516484	11.6010989	1014.992857	1010.214835	1012.651648	79.23076923	5.215934066	8.284615385
	10724	327	22.82252747	15.37802198	18.34725275	1011.895055	1006.754945	1009.424176	81.66483516	4.799450549	7.537362637
	10724	328	17.13131868	13.69340659	14.96263736	1011.118132	1007.026923	1009.117033	86.67582418	5.012087912	8.175274725
	10724	329	17.4	13.08241758	14.92802198	1014.051648	1008.197253	1011.174176	82.16574586	4.941758242	8.048351648
	10724	330	22.86923077	15.51923077	18.31648352	1011.667582	1006.418132	1009.185714	78.71823204	5.001098901	8.006043956
	10724	331	26.73021978	17.66098901	21.42362637	1008.442308	1003.114835	1005.935714	77.03296703	4.945604396	7.713186813
	10724	332	24.16923077	18.27087912	20.56923077	1006.312637	1000.99011	1003.607143	85.95604396	6.170879121	10.16593407
	10724	333	25.84120879	19.51483516	22.01208791	1007.696703	1003.22033	1005.578571	83.32417582	4.528021978	7.123076923
	10724	334	28.4032967	21.64505495	24.46263736	1005.865934	1001.525824	1003.821978	78.12087912	4.912087912	7.737362637
	10724	335	26.84835165	22.02527473	23.91648352	1004.98956	1000.747253	1002.944505	85.51098901	4.996153846	7.908241758
	10724	336	26.86098901	20.11428571	22.81538462	1006.946703	1002.97033	1005.02033	80.37362637	4.725274725	7.462637363
	10724	337	24.33021978	19.84010989	21.58351648	1005.593956	1000.556044	1003.182967	90.17582418	4.802747253	7.749450549
	10724	338	30.08021978	24.02857143	26.31263736	999.9395604	995.5005495	997.9417582	88.57142857	5.695604396	9.291208791
	10724	339	30.17802198	24.08626374	26.39945055	1000.891758	996.867033	999.0813187	88.68131868	5.995054945	10.06703297
	10724	340	32.89065934	25.33241758	28.23461538	1001.355495	997.1379121	999.4807692	83.45604396	5.678571429	9.182417582
	10724	341	32.14725275	25.44340659	28.09285714	997.2505495	993.2692308	995.4631868	83.60989011	5.384615385	8.816483516
	10724	342	32.29945055	24.60659341	27.81373626	995.1791209	991.8357143	993.7098901	77.18681319	4.805494505	7.715384615
	10724	343	32.81373626	26.01428571	28.73406593	995.8428571	992.0087912	994.2318681	82.97802198	5.885714286	9.628571429
2014	10724	344	32.27692308	25.85	28.42857143	997.5263736	994.0203297	996.0043956	85.1978022	5.029120879	8.2
	10724	345	33.54120879	26.44395604	29.40054945	998.6368132	994.7324176	996.9115385	79.85164835	5.298351648	8.565934066
	10724	346	34.45494505	26.11758242	29.36428571	996.4362637	992.6697802	994.8450549	80.2032967	5.60989011	8.981318681
	10724	347	33.27362637	26.05824176	29.16318681	1000.337363	995.4351648	998.3862637	78.60989011	6.503846154	10.88516484
	10724	348	34.22747253	25.61043956	29.0956044	997.9357143	992.95	995.8307692	79.53846154	5.613186813	9.204395604
	10724	349	34.79120879	25.56813187	29.37032967	997.8054945	993.0456044	995.8362637	76.42857143	5.123626374	8.137912088
	10724	350	34.11483516	25.99889503	29.20824176	995.9093407	992.078022	994.260989	79.57692308	5.458241758	8.621428571
	10724	351	32.37637363	25.66243094	28.20549451	998.4950549	994.1637363	996.6098901	84.42857143	5.950549451	9.686263736
	10724	352	31.75714286	24.48736264	27.38956044	1001.756044	998.2489011	1000.270879	84.49450549	5.387362637	8.598901099
	10724	353	33.48626374	24.91263736	28.44395604	1003.857692	999.9362637	1002.222527	78.92857143	5.388461538	8.794505495
	10724	354	33.6989011	24.86318681	28.36648352	1002.246154	998.1241758	1000.336264	79.36813187	5.213736264	8.347802198
	10724	355	33.12307692	25.65054945	28.60494505	1000.402747	996.5274725	998.6934066	81.47802198	4.868131868	7.802747253
	10724	356	32.30494505	25.2467033	28.05	1001.069231	994.9747253	998.2725275	81.19230769	6.187912088	10.45494505
	10724	357	31.70384615	23.59450549	26.96373626	1003.30989	999.7989011	1001.591209	73.96153846	4.216483516	6.776373626
	10724	358	32.7467033	24.29065934	27.70494505	1004.742308	1000.428571	1002.642308	77.53296703	4.548901099	7.31043956
	10724	359	30.22472527	20.31923077	24.75714286	1006.826923	1002.283516	1004.588462	61.28021978	5.047802198	8.362087912
	10724	360	28.97857143	19.53241758	23.61703297	1010.768681	1005.936264	1008.409341	64.32417582	4.851648352	7.907692308
	10724	361	29.36538462	21.05989011	24.46868132	1009.375275	1005.353297	1007.291209	73.36263736	4.388461538	7.074175824
	10724	362	29.97087912	21.61208791	24.99505495	1008.787363	1004.76044	1006.814835	75.21978022	4.438461538	7.151098901
	10724	363	23.88076923	18.62252747	20.64175824	1011.287363	1006.885714	1009.120879	76.64285714	4.97967033	8.089010989
	10724	364	22.37252747	17.28131868	19.27967033	1012.624725	1019.531868	1010.568681	80.52747253	4.526373626	7.285164835
	10724	365	24.73296703	15.82142857	19.49010989	1012.995604	1008.147802	1010.653297	71.64285714	4.764285714	7.651098901
	10724	366	27.15879121	19.11428571	22.22747253	1008.095604	1003.551099	1005.809341	81.73076923	4.298901099	6.918681319
	10724	367	19.77197802	13.52967033	16.00274725	1013.128022	1007.676374	1010.616484	75.67582418	5.721978022	9.372527473
	10724	368	18.89395604	12.0532967	14.73626374	1016.151099	1011.357692	1013.843956	67.24175824	5.546703297	8.978571429
	10724	369	17.32857143	8.817582418	12.43461538	1018.696154	1012.816484	1015.953297	56.73626374	5.290659341	8.652747253
	10724	370	17.61593407	10.5456044	13.53296703	1016.051648	1011.259341	1013.692857	71.10989011	4.985164835	8.101098901
	10724	371	18.74230769	7.019230769	11.64134615	1015.842308	1010.3	1013.021154	71.56730769	4.771153846	7.596153846

	10849	372	20.01162791	7.607751938	12.60310078	1018.440698	1012.568992	1015.585271	62.94573643	4.310077519	6.864341085
	10849	373	20.16096346	11.71312292	15.01877076	1014.287209	1009.15814	1011.782558	73.535	4.362292359	6.940199336
	10849	374	17.16644518	8.73089701	12.11627907	1017.101495	1012.416113	1014.705648	76.63666667	4.865946844	7.878903654
	10849	375	21.07325581	8.654983389	13.7986711	1015.470764	1010.351993	1012.857143	68.18936877	4.293023256	6.791860465
	10849	376	19.79883721	13.06196013	15.53006645	1013.936379	1009.376246	1011.645847	80.15282392	4.381530782	6.903327787
	10849	377	16.58023256	9.694352159	12.40863787	1018.866279	1014.330731	1016.632724	72.9833887	4.485049834	7.181063123
	10849	378	19.64833887	9.620431894	13.85930233	1014.432226	1009.56794	1011.917442	64.92358804	4.225415282	6.66461794
	10849	379	23.17524917	15.98853821	18.65797342	1010.698007	1006.246512	1008.464452	83.44518272	4.271096346	6.783056478
	10849	380	24.53704319	18.32641196	20.69202658	1008.133389	1003.226412	1005.772591	86.3986711	5.046511628	8.041528239
	10849	381	17.36727575	12.46212625	14.38006645	1011.32907	1005.566445	1008.485548	86.23255814	4.689700997	7.498671096
	10849	382	19.44169435	14.26578073	16.29501661	1013.325415	1008.946844	1011.162791	87.13122924	4.06422629	6.406156406
	10849	383	26.37342193	20.11179402	22.63936877	1007.508306	1002.590199	1005.160631	86.18604651	4.573089701	7.249667774
	10849	384	23.05	17.21129568	19.50415282	1014.777741	1010.212458	1012.527243	79.6013289	4.294850498	6.769435216
	10849	385	29.06495017	21.62126246	24.66129568	1004.549834	999.4747508	1002.083223	80.15946844	5.442192691	8.776744186
	10849	386	23.02342193	16.73920266	19.1961794	1009.799834	1005.498173	1007.802159	83.1744186	4.876910299	7.844684385
	10849	387	27.7282392	15.21312292	21.03056478	1009.442857	1003.88505	1006.920432	67.28571429	5.132225914	8.358139535
	10849	388	27.5551495	20.10431894	23.10963455	1007.686379	1003.196179	1005.668439	77.25913621	4.850166113	7.930398671
	10849	389	29.94833887	22.21129568	25.29816972	1005.246844	1000.618439	1003.014975	81.58402662	4.913289037	7.88654485
	10849	390	29.73122924	23.69451827	25.9089701	1002.128738	997.8815615	1000.131894	88.18272425	5.198006645	8.463953488
	10849	391	30.06827243	22.48156146	25.64451827	1004.242193	999.9181063	1002.217442	81.76245847	5.558139535	9.146843854
	10849	392	28.53887043	23.64950166	25.48289037	1000.347342	996.6755814	998.591196	91.5448505	5.019435216	8.319601329
	10849	393	32.08903654	25.16179402	27.81511628	999.3277409	995.4501661	997.6112957	86.90365449	5.329568106	8.855481728
	10849	394	32.34501661	24.86993355	27.63621262	1001.022425	997.3662791	999.338206	85.4833887	6.380066445	10.74850498
	10849	395	33.03322259	25.34368771	28.22475083	1000.587874	996.6750831	998.9282392	83.54817276	6.479568106	11.01843854
	10849	396	34.53953488	25.86877076	29.57209302	999.7355482	995.9659468	998.1021595	77.02159468	5.433388704	8.845348837
	10849	397	32.74418605	26.11395349	28.73936877	998.019103	994.6408638	996.4998339	82.97508306	5.730116473	9.594509151
2015	10849	398	34.94750831	27.31328904	30.64186047	997.6933555	993.4571429	995.8466777	75.56644518	5.255315615	8.525083056
	10849	399	31.16212625	24.39385382	27.19451827	994.9435216	991.1684385	993.2803987	76.76206323	5.549833887	9.144518272
	10849	400	34.56461794	26.03222591	29.48671096	994.6171096	990.9327243	993.1093023	78.70265781	4.748419301	7.613643927
	10849	401	30.9666113	25.25780731	27.38803987	998.2506645	994.8813953	996.7682724	87.24916944	5.931893688	10.09435216
	10849	402	32.19352159	24.34767442	27.51131448	1003.446346	1000.173588	1002.00814	81.19269103	5.748504983	9.51910299
	10849	403	34.94800664	24.99534884	29.37259136	1000.239535	995.4420266	998.1159468	72.66611296	4.518469218	7.377740864
	10849	404	32.4307309	25.31478405	27.83272425	998.7466777	994.7916944	997.0710963	84.56644518	5.852159468	9.733388704
	10849	405	33.84069767	25.81910299	29.01710963	999.4350498	995.1405316	997.4774086	80.55647841	4.84269103	7.903322259
	10849	406	33.55016611	25.05016611	28.46212625	998.7483389	995.0172757	997.095515	76.1179402	4.746677741	7.561461794
	10849	407	30.16196013	24.46511628	26.53693844	1002.907973	999.4840532	1001.373544	89.85856905	4.382392027	7.171594684
	10849	408	32.16196013	24.55797342	27.55598007	1003.551993	999.4122924	1001.7701	80.48006645	4.480398671	7.155813953
	10849	409	31.47043189	23.58737542	26.88803987	1006.768605	1002.392525	1004.76173	76.2063228	4.558305648	7.350332226
	10849	410	31.55415282	24.56561462	27.29767442	1001.453156	997.1523256	999.4883721	84.79900332	4.196345515	6.729734219
	10849	411	31.34534884	23.90598007	26.9910299	1004.17392	999.7805648	1002.221096	75.76910299	4.835714286	7.911461794
	10849	412	28.07209302	23.06112957	25.02607973	1006.993023	1002.54103	1004.824751	84.74086379	5.366112957	9.000498339
	10849	413	28.18372093	18.2833887	22.47607973	1010.822924	1006.248671	1008.674252	71.32890365	4.068438538	6.651162791
	10849	414	30.18156146	19.16196013	23.89119601	1006.002159	1001.430731	1003.714452	70.97840532	3.965946844	6.492857143
	10849	415	28.74302326	21.80448505	24.45415282	1011.184053	1007.044684	1009.119601	81.4551495	4.240365449	6.709136213
	10849	416	26.60647841	18.87275748	22.02342193	1012.485548	1008.107143	1010.232724	78	4.182225914	6.720431894
	10849	417	25.25930233	19.68887043	21.78604651	1010.562458	1006.578073	1008.504485	85.15806988	4.356976744	6.921096346
	10849	418	28.17940199	21.14966777	23.89053156	1009.230897	1005.11794	1007.178405	84.87873754	4.223255814	6.70448505
	10849	419	23.74136213	15.28386023	18.78853821	1013.947508	1009.254651	1011.677409	70.89368771	5.061129568	8.199003322
	10849	420	20.97973422	15.19285714	17.41179402	1013.887043	2653.78289	1011.568386	83.10299003	4.503322259	7.27076412
	10849	421	17.36860465	12.78471761	14.64800664	1014.113289	1009.242359	1011.625748	84.30730897	4.447508306	7.24833887
	10849	422	18.03853821	10.76046512	13.66422629	1016.368439	1011.989369	1014.307155	67.85191348	5.040365449	8.382392027
	10849	423	20.0692691	14.05398671	16.43671096	1014.539535	1009.581063	1012.04701	84.83554817	4.017607973	6.391196013
	10849	424	17.32093023	12.19389535	14.28633721	1019.453779	1015.034884	1017.370349	81.93895349	4.308139535	6.925

	10906	425	21.49069767	11.69186047	15.72517442	1018.68314	1012.90814	1015.723314	79.68604651	3.20872093	5.00755814
	10906	426	21.35431894	15.74451827	17.97885593	1013.421429	1008.4299	1010.691716	85.1371822	4.054817276	6.402325581
	10906	427	16.36262458	12.45398671	14.0025	1012.184219	1007.102658	1009.67	86.18577586	4.3	6.95
	10906	428	14.22757475	9.835215947	11.55764103	1014.096346	1008.379402	1011.484726	82.83803419	5.2589701	8.78986711
	10906	429	12.48289037	6.989700997	8.570718447	1020.194684	1013.702326	1017.608058	77.14854369	4.926245847	8.211627907
	10906	430	14.02275748	8.02076412	10.35694352	1017.378738	1012.02309	1015.155923	75.77616279	5.515282392	9.16179402
	10906	431	22.79186047	10.6166113	15.66122924	1011.940698	1006.450997	1009.267492	72.5967608	4.605149502	7.515946844
	10906	432	16.65780731	10.32059801	12.70765781	1016.81113	1010.536213	1014.002674	71.7595515	4.854817276	8.009302326
	10906	433	14.77142857	10.97973422	12.47438655	1019.101827	1014.387708	1016.708067	83.7210084	4.281561462	6.947009967
	10906	434	23.8127907	11.08754153	16.44813953	1015.266777	1010.126246	1012.774934	75.21968439	4.109468439	6.501827243
	10906	435	19.39850498	13.4448505	15.77923461	1010.904817	1004.643023	1007.971248	86.26830283	4.625913621	7.496345515
	10906	436	18.22890365	14.13671096	15.94830565	1008.345515	1003.810797	1006.067126	91.76951827	3.67192691	5.89717608
	10906	437	20.67973422	14.92142857	17.10096506	1010.98289	1005.509635	1008.500433	86.75374376	5.392857143	9.011129568
	10906	438	23.81461794	15.48887043	18.42606589	1012.47309	1007.521096	1010.655601	73.47383721	3.977574751	6.286378738
	10906	439	27.45697674	21.26528239	23.75167774	1005.619269	1000.928738	1003.410183	87.06353821	4.964950166	8.136212625
	10906	440	26.30498339	21.37392027	23.28662791	1001.877907	997.0956811	999.5319934	90.59011628	5.302325581	9.037209302
	10906	441	26.38355482	20.61245847	22.85001661	1005.981395	1000.363123	1003.420299	88.285299	5.536710963	9.316611296
	10906	442	28.20182724	21.9910299	24.36574751	1003.52608	999.3284053	1001.517691	83.88372093	4.512624585	7.343355482
	10906	443	30.39900332	22.87607973	25.990299	1003.322093	999.0024917	1001.367807	86.11669435	5.49551495	9.098172757
	10906	444	29.99053156	23.00631229	25.9734609	1003.631728	999.3004983	1001.550849	82.25915141	4.737043189	7.78538206
	10906	445	28.19451827	21.70614618	24.40425249	1003.628738	999.1059801	1001.500449	80.94352159	4.928239203	8.157641196
	10906	446	30.96079734	24.13122924	26.98171667	1000.664618	996.8380399	999.0474333	82.02958333	4.768438538	7.818272425
	10906	447	33.98887043	25.90348837	29.10238333	1000.111462	995.8965116	998.2366167	82.57041667	6.038039867	10.1166113
	10906	448	31.3730897	24.48189369	26.95931894	999.8548173	996.166113	998.2533223	87.91943522	4.829900332	7.881395349
	10906	449	31.92059801	25.33106312	27.82273032	998.8074751	995.2290698	997.2361681	88.34175084	5.591694352	9.510465116
	10906	450	34.65299003	25.93388704	29.89968439	1001.615947	997.6373754	999.895814	76.66818937	5.021096346	8.143687708
2016	10906	451	33.14368771	25.93006645	28.8396	1001.687708	998.0146179	1000.04455	83.41708333	5.672259136	9.731727575
	10906	452	33.96976744	25.72408638	29.06769103	998.7762458	994.429402	996.682309	81.46345515	5.609634551	9.599501661
	10906	453	32.10681063	25.489701	27.85237542	996.948505	993.8372093	995.5030399	88.36129568	5.550664452	9.289534884
	10906	454	34.29916944	26.30066445	29.74843594	1001.383721	997.3855482	999.6258472	77.84219269	5.263289037	8.867275748
	10906	455	35.80365449	25.79601329	30.03539101	1001.220432	997.4068106	999.5609983	74.6859401	5.002990033	8.281395349
	10906	456	33.38554817	25.25664452	28.36028286	998.5302326	992.2945183	995.8130283	83.82695507	5.842026578	9.921428571
	10906	457	32.9333887	25.89003322	28.36076539	995.3599668	991.3446844	993.5353156	86.93936877	4.467774086	7.321096346
	10906	458	31.45747508	25.41960133	27.59136213	992.7107973	987.9335548	990.5103821	88.4788206	5.647342193	9.796179402
	10906	459	34.18289037	25.79019934	29.01661667	997.7745847	993.8689369	996.0103993	83.48169717	4.776744186	7.869269103
	10906	460	31.24086379	24.4333887	27.00634551	998.4727575	994.607309	996.6628239	83.56727575	4.477574751	7.392358804
	10906	461	31.28255814	25.20930233	27.19438538	999.7034884	996.5827243	998.288505	89.45390365	4.485049834	7.277242525
	10906	462	32.16960133	24.62790698	27.72207641	1000.85897	996.6694352	998.7676578	79.15905316	4.717940199	7.87358804
	10906	463	31.69003322	23.40265781	26.97744186	1004.553654	1000.707807	1002.788883	73.68230897	4.44717608	7.303820598
	10906	464	31.06362126	23.28089701	26.40163062	1000.373754	995.3533223	997.9816805	77.76039933	4.795681063	8.079734219
	10906	465	31.33754153	23.84318937	26.78526578	1002.336379	998.3611296	1000.45485	80.44352159	4.435548173	7.31744186
	10906	466	27.74950166	21.46843854	24.08480066	1005.991196	1002.304153	1004.148588	76.20182724	4.932890365	8.265946844
	10906	467	29.48920266	23.33006645	25.79127907	1001.706146	995.6141196	998.9728571	84.18729236	5.942857143	10.13438538
	10906	468	30.61262458	23.39318937	26.03936877	1007.801993	1003.151163	1005.416988	85.28322259	3.928737542	6.36345515
	10906	469	25.53787375	17.68289037	20.91451827	1012.721761	1007.918605	1010.48299	72.52574751	4.793023256	7.965448505
	10906	470	23.35614618	16.27807309	19.11686047	1012.251329	1007.818937	1010.02211	82.86503322	4.258471761	7.073089701
	10906	471	28.90548173	20.76046512	23.85709302	1008.89103	1004.846512	1006.825349	85.61669435	3.449833887	5.565282392
	10906	472	20.42275748	15.66445183	17.59913621	1010.440033	1005.533555	1007.920598	89.75290698	5.065946844	8.469601329
	10906	473	21.08504983	11.97873754	15.83096346	1016.087375	1011.266611	1013.722326	73.17026578	4.612292359	7.64717608
	10906	474	23.94518272	13.75797342	17.75564784	1012.21711	1007.22691	1009.803306	71.11295681	3.926910299	6.281229236
	10906	475	21.39152824	12.92093023	16.3419103	1013.806645	1008.656645	1011.420648	72.7666113	4.92641196	8.120099668
	10906	476	23.39186047	15.46229236	18.62345515	1012.552824	1007.565615	1010.130332	77.97425249	4.11744186	6.640199336
	10906	477	19.86744186	11.10681063	14.52159468	1016.149169	1010.986379	1013.70814	69.75664452	4.9910299	8.429568106
